# From gut to spinal cord glymphatic: Ginkgolide B’s multifaceted approach to alleviating painful diabetic neuropathy

**DOI:** 10.3389/fmicb.2026.1833646

**Published:** 2026-05-13

**Authors:** Shuai-ying Jia, Pin-xiu Chen, Jun-lin Wang, Wan-xin Liu, Jing-ting Wang, Wen-qin Yin, Jing-yan Lin

**Affiliations:** 1 Department of Anesthesiology, The Affiliated Hospital of North Sichuan Medical College, Nanchong, China; 2 Department of Anesthesiology, Nanchong Hospital of Traditional Chinese Medicine, Nanchong, China

**Keywords:** aquaporin-4, Ginkgolide B, glymphatic system, gut microbiota, painful diabetic neuropathy

## Abstract

**Background:**

Painful diabetic neuropathy (PDN) is a common complication of type 2 diabetes, characterized by neuropathic pain and inflammation. Its pathogenesis involves oxidative stress, inflammatory responses, and dysfunction of the spinal cord glymphatic system. This study aimed to investigate the protective effects of Ginkgolide B (GB) in alleviating PDN, with a particular focus on its roles in modulating the gut microbiota and enhancing glymphatic function in the spinal cord.

**Methods:**

A PDN model was established in male Sprague–Dawley rats to evaluate the therapeutic effects of GB. GB was administered to assess its impact on gut microbiota composition, intestinal barrier integrity, and inflammation in both the intestine and spinal cord. Additionally, the effect of GB on aquaporin-4 (AQP4) polarization in the spinal cord glymphatic system was examined to determine its role in facilitating the clearance of inflammatory mediators.

**Results:**

GB treatment significantly alleviated hallmark features of PDN, including neuropathic pain and spinal cord inflammation. It modulated the gut microbiota, restored intestinal barrier function, and reduced intestinal inflammation. Moreover, GB reestablished AQP4 polarity in the spinal cord, thereby enhancing glymphatic function and promoting the clearance of inflammatory mediators, which contributed to reduced neuroinflammation.

**Conclusion:**

These findings suggest that Ginkgolide B may represent a multifaceted therapeutic strategy for PDN. By regulating the microbiota–gut–spinal cord glymphatic axis, improving glymphatic function, and alleviating PDN symptoms, GB shows promise as a novel treatment targeting both metabolic and neuroinflammatory components of the disease.

## 1 Introduction

Type 2 diabetes mellitus (T2DM) is a chronic metabolic disorder with a rapidly increasing global prevalence. Painful diabetic neuropathy (PDN), affecting approximately 20–30% of patients with diabetes, is among its most debilitating complications (Sun et al., 2022; Aronson et al., 2021). Clinically, PDN is characterized by symmetrical sensory disturbances and neuropathic pain in the lower extremities, including burning sensations, electric shock–like pain, hyperalgesia, and allodynia (Sloan et al., 2018). Its pathogenesis is multifactorial, involving hyperglycemia-induced oxidative stress, neuroinflammation, and microvascular dysfunction, ultimately leading to injury of both the peripheral and central nervous systems (Feldman et al., 2019). Current therapeutic strategies primarily focus on glycemic control and symptomatic pain relief; however, they fail to effectively address the underlying pathogenic mechanisms (Tesfaye et al., 2022). Therefore, identifying novel mechanistic targets for PDN is of significant clinical importance.

The glymphatic system, a recently characterized cerebrospinal fluid–interstitial fluid exchange pathway, plays a crucial role in maintaining central nervous system (CNS) homeostasis by facilitating the clearance of metabolic waste (Hablitz and Nedergaard, 2021; Iliff et al., 2012). Analogous structures have also been identified in the spinal cord (Liu et al., 2018). Aquaporin-4 (AQP4), predominantly localized to astrocytic end-feet in a highly polarized distribution, is essential for glymphatic function (Iliff et al., 2012). Disruption of AQP4 polarity impairs solute clearance and has been implicated in various neurological disorders (Iliff et al., 2012; Kress et al., 2014; Peng et al., 2023). Notably, alterations in AQP4 polarity have been observed under pathological conditions, including diabetic neuropathy (Li et al., 2024; Jia et al., 2024). However, the upstream regulatory mechanisms governing AQP4 polarity in PDN remain largely unclear.

Emerging evidence indicates that gut microbiota dysbiosis contributes to metabolic disorders and diabetic complications, including neuropathy (Iatcu et al., 2021). Both clinical and experimental studies have demonstrated alterations in microbial composition in patients with PDN, and fecal microbiota transplantation from healthy donors has been shown to alleviate neuropathic symptoms independently of glycemic control (Wang et al., 2020; Yang et al., 2023; Sabico et al., 2019). Mechanistically, gut microbiota–derived metabolites, particularly short-chain fatty acids (SCFAs), can modulate neuroinflammation and preserve AQP4 polarity in an aryl hydrocarbon receptor (AhR)–dependent manner, thereby maintaining blood–brain barrier integrity and glymphatic function (Salminen, 2023; Lin et al., 2024). These findings suggest that the gut microbiota may influence spinal cord glymphatic function through regulation of AQP4 polarity. However, whether a gut–spinal cord glymphatic axis exists in PDN and contributes to neuropathic pain remains to be elucidated.

Ginkgolide B (GB), a terpene lactone derived from *Ginkgo biloba*, exhibits anti-inflammatory, antioxidant, and neuroprotective properties (DeFeudis and Drieu, 2000; Huang et al., 2021; Yang et al., 2021). Recent studies have shown that GB can modulate gut microbiota composition and improve metabolic and inflammatory status (Liu et al., 2021; Lv et al., 2021). Although GB has demonstrated protective effects in experimental models of PDN (Li et al., 2024), it remains unclear whether its therapeutic benefits involve regulation of the gut microbiota and subsequent restoration of spinal cord glymphatic function.

Therefore, this study aimed to determine whether GB alleviates PDN by modulating gut microbiota composition and restoring AQP4-dependent glymphatic function in the spinal cord. By elucidating the potential gut–spinal cord glymphatic axis in PDN, this work seeks to provide mechanistic insight into a novel therapeutic strategy for diabetic neuropathic pain.

## 2 Methods and materials

### 2.1 Animals

Eighty male Sprague–Dawley (SD) rats (6–8 weeks old, weighing 160–180 g) were obtained from the Animal Center of North Sichuan Medical College. All animals were housed individually under specific pathogen-free conditions, maintained at a temperature of 24 ± 1 °C, relative humidity of 55 ± 5%, and a 12 h light–dark cycle. Rats were provided with irradiated sterilized chow and reverse osmosis–purified sterile water ad libitum. All experimental procedures were approved by the Animal Ethics Committee of North Sichuan Medical College (Approval No.: NSMC 2024[071]).

### 2.2 PDN rat model

The procedure was performed as previously described streptozotocin-induced diabetic models in mice and rats, with minor modifications (Furman, 2021). Briefly, all rats were acclimated for 1 week prior to the experiment. Subsequently, 80 rats were randomly assigned to the model group (*n* = 70) and the control group (group C, *n* = 10). During the entire experimental period, rats in group C were fed a standard maintenance diet sterilized by irradiation. In contrast, rats in the model group were fed a high-fat and high-sugar diet (10% sucrose, 10% lard, and 5% cholesterol; Xiaoshuyoutai Biotechnology Co., Ltd., Beijing, China; product number D12450J) for 4 weeks to induce insulin resistance. After 4 weeks of dietary intervention, type 2 diabetes mellitus was induced in the model group by intraperitoneal injection of low-dose streptozotocin (STZ; HY-13753, MedChemExpress, Monmouth Junction, NJ, United States). STZ was dissolved in 10 mmol/L citrate buffer (pH 4.5) and administered at a dose of 35 mg/kg with an injection volume of 1.0 mL/kg. Twenty-four hours after injection, fasting blood glucose levels were measured via tail vein sampling. Rats with FBG levels >16.7 mmol/L were considered diabetic. Rats that did not meet this criterion received additional STZ injections at the same dose, with a maximum of three injections. Animals that failed to reach the diabetic threshold after three injections were excluded. This procedure has been employed in numerous diabetes-related studies (Li et al., 2024; Jia et al., 2024). Rats with confirmed hyperglycemia underwent intraperitoneal glucose tolerance tests (IPGTT) and insulin tolerance tests (ITT) to evaluate glucose metabolism and insulin resistance. In addition, 24-h food and water intake were recorded to assess polyphagia and polydipsia. Mechanical hypersensitivity was assessed using the paw withdrawal threshold (PWT). Diabetic rats exhibiting both persistent hyperglycemia and a significant reduction in PWT compared with the control group were considered to have developed PDN. Only rats meeting these criteria were included in subsequent experiments. Rats that did not meet the PDN criteria were euthanized by intraperitoneal injection of 2% pentobarbital in accordance with ethical guidelines. Finally, a total of 32 rats with confirmed PDN were included in subsequent experiments.

### 2.3 Antibiotic pseudaseptic PDN model

Prior to oral gavage treatment, a subset of PDN rats received an antibiotic cocktail (ABX) in their drinking water, consisting of ampicillin (1 g/L), neomycin (1 g/L), metronidazole (1 g/L), and vancomycin (0.5 g/L) for 1 week (Ren et al., 2025). Thereafter, these rats were randomly assigned to either the GB-treated pseudogerm-free PDN group (group PDN + ABX + GB) or the untreated pseudogerm-free PDN group (group PDN + ABX).

### 2.4 Animal grouping and treatment protocols

Rats were randomly assigned to the following experimental groups:

Group C (Healthy control, *n* = 10): Healthy rats received an equivalent volume of saline via oral gavage once daily for 21 consecutive days.

Group PDN (PDN, *n* = 10): PDN rats were administered an equivalent volume of saline by gavage following the same schedule.

Group PDN + GB (*n* = 10): PDN rats received Ginkgolide B (GB; CAS No. 15291-77-7; Alfa Biology, Chengdu, China) at a dose of 10 mg/kg by oral gavage once daily for 21 consecutive days. This dosage and administration regimen were selected based on our previous study and preliminary experiments, which demonstrated significant therapeutic efficacy at this dose (Li et al., 2024).

Group PDN + ABX (pseudogerm-free PDN, *n* = 6): PDN rats were rendered pseudogerm-free by antibiotic (ABX) treatment and subsequently received sterile water by gavage once daily for 21 days.

Group PDN + ABX + GB (pseudogerm-free PDN + GB, *n* = 6): Pseudogerm-free PDN rats received Ginkgolide B (10 mg/kg) by oral gavage once daily for 21 days.

Throughout the experiment, body weight and fasting blood glucose levels were recorded weekly. Upon completion of the gavage treatment, all rats underwent magnetic resonance imaging (MRI) scanning. Following imaging, fecal samples, spinal cord tissues, and colon tissues were collected for subsequent analyses. The experimental timeline is illustrated in Supplementary Figure S1.

### 2.5 Intraperitoneal glucose tolerance and insulin tolerance experiments

In week 7, rats in the model group exhibiting elevated blood glucose levels and significantly reduced paw withdrawal thresholds (PWT) were selected for further analysis. These animals, along with control rats, underwent intraperitoneal glucose tolerance testing (IPGTT) and insulin tolerance testing (ITT) to evaluate glucose metabolism and insulin sensitivity.

#### 2.5.1 Intraperitoneal glucose tolerance test

Rats were fasted for 12 h prior to testing, with free access to water. Fasting blood glucose levels were measured via tail vein sampling to establish baseline values. Subsequently, a 50% glucose solution (2 g/kg) was administered intraperitoneally. Blood glucose concentrations were measured at 30, 60, 90, and 120 min post-injection to assess glucose tolerance (Watada et al., 2014).

#### 2.5.2 Insulin tolerance test

Forty-eight hours after IPGTT, ITT was performed. Baseline blood glucose levels were measured prior to insulin administration. Rats were then intraperitoneally injected with insulin (NovoRapid, Novo Nordisk, Denmark) at a dose of 0.5 U/kg. Blood glucose levels were recorded at 30, 60, 90, and 120 min post-injection. Insulin sensitivity was evaluated by calculating the percentage change in blood glucose relative to baseline, allowing assessment of insulin resistance and its severity (Dolo et al., 2018).

### 2.6 Food and water intake measurement

Food and water intake were monitored over 24 h during week 7 to assess metabolic changes in diabetic rats. Measurements were conducted on days 1, 4, and 7. During each assessment, rats were housed individually with ad libitum access to food and water. The initial weights of food and water were recorded, and the remaining amounts were measured after 24 h. Intake was calculated as the difference between pre- and post-measurement weights. To account for inter-individual differences in body weight, intake values were normalized and expressed per 100 g body weight (g/100 g BW), calculated by dividing the 24 h intake (g) by the body weight (g). All measurements were performed by a trained investigator blinded to group allocation.

### 2.7 Paw withdrawal threshold detection

In this study, PWT, a well-established measure of mechanical hypersensitivity, was used to evaluate nociceptive responses in rats. Assessments commenced in the fifth week and were performed weekly by a trained investigator using a von Frey filament kit (US PAT. 58239698512259, North Coast, CA, United States). The testing procedure was conducted as follows: von Frey filaments were applied perpendicularly to the central plantar surface of the hind paw with controlled force. A positive response was defined as paw withdrawal, licking, or other distinct nocifensive behaviors. If at least three positive responses were observed in five consecutive applications, the corresponding filament force was recorded as the PWT (Su et al., 2019). Prior to testing, rats were placed on a wire mesh platform and allowed to acclimate for 15 min to s and an inter-stimulus interval of approximately 30s (Tokhi et al., 2023). To prevent tissue damage, the maximum applied force did not exceed 26 g.

### 2.8 Magnetic resonance imaging

MRI-based glymphatic function assessment was performed using a tracer-based method adapted from previous studies (Qi et al., 2018). Although glymphatic studies are primarily performed in the brain, this approach was adapted here to evaluate spinal glymphatic-like transport. After the treatment period, four rats from each group were randomly selected for MRI scanning. The experiment was conducted in the MRI room at the Women and Children’s Hospital of North Sichuan Medical College, using a 3.0 T MRI system (Discovery MR750, GE, United States). The rats were anesthetized with isoflurane and placed in an eight-channel dedicated rat coil. The anesthesia was maintained using a mixture of 2.5–3.0% isoflurane and oxygen (2.5 L/min). The scanning region was centered on the 13th thoracic vertebra of the rat, extending 2.5 cm upward and downward. T1-weighted Fast Spin Echo (FSE) sequences were used for imaging to observe the distribution of the contrast agent in the spinal cord. The scanning parameters were as follows: repetition time (TR) 450.0 ms, echo time (TE) 5.4 ms, slice thickness 2.0 mm, slice gap 1.0 mm, field of view (FOV) 6.5 cm × 6.5 cm, matrix size 256 × 256, flip angle 90°, bandwidth 31.25 kHz, and frequency direction: anterior/posterior (A/P) (Li et al., 2024; Jia et al., 2024). After acquiring the initial images, the rats were positioned prone on the operating table, with a cushion placed under the abdomen for stability. The operation method is consistent with that of previous studies (Li et al., 2024; Jia et al., 2024). Specifically, the back hair was shaved and disinfected. The L6 vertebra was used as the landmark for performing a lumbar puncture using a 50 μL syringe (Gaoge, China). Upon successful puncture, the rats exhibited rapid lateral tail movement. Subsequently, 25 μL of 10% Gadopentetic acid (Gd-DTPA, prepared by dissolving 100 μL of Gd-DTPA in 900 μL of sterile saline) was injected into the subarachnoid space at the L4/5 level. The injection rate was consistent, and the injection duration lasted 5 min. After injection, the needle was kept in place for 3 min to prevent reflux, and then the needle was removed, and the rat was returned to the MRI coil for scanning. Following the contrast agent injection, MRI scans were performed at 5 min, 15 min, 30 min, 1 h, 1.5 h, 2 h, 2.5 h, and 5 h post-injection to assess tracer influx and clearance dynamics within the spinal cord. Throughout the experiment, the rats’ vital signs were continuously monitored to ensure their heart rate remained between 250 and 450 beats per minute, and their body temperature was maintained between 36.5 °C and 37.5 °C using a feedback-controlled air heating fan. During scan intervals, the rats were allowed to drink freely once they regained consciousness. For quantitative analysis, the largest cross-sectional image of the spinal cord and adjacent slices were selected. Two blinded evaluators used RadiAnt DICOM Viewer (64 bit; Version 2021.1, Medixant, Poland) to measure the MRI signal intensity (SI) of the spinal cord gray matter. For each rat, SI values were averaged from three measurements within a fixed region of interest (approximately 0.04 cm^2^) covering most of the gray matter. The MRI SI reflects the distribution and clearance of the intrathecally injected tracer and was used as an indirect indicator of glymphatic transport function. The SIPH value, which reflects the rate of change in signal intensity, was calculated using the formula: SIPH = (Mean(PEAK SI) - Mean(SIX SI))/(6- Mean(PEAK TIME)).

### 2.9 Histopathology

Rats were deeply anesthetized with 2% pentobarbital, and blood was cleared by transcardiac perfusion with phosphate-buffered saline (PBS). Subsequently, spinal cord and colon tissues were harvested and fixed in 4% paraformaldehyde. The fixed tissues were paraffin-embedded and sectioned into 4–6 μm thick slices. Hematoxylin and eosin (H&E) staining was performed following standard protocols to ensure consistency and reproducibility. Histological changes were examined under a light microscope. For histopathological evaluation of colonic injury, a semi-quantitative scoring system was applied based on a previously published method (Rakoff-Nahoum et al., 2004). All sections were evaluated in a blinded manner by trained investigators to minimize bias. The following parameters were assessed: inflammatory cell infiltration, submucosal edema, loss of goblet cells, and crypt epithelial hyperplasia. Each of these features was graded on a scale of 0 to 3 as follows: 0 = normal, 1 = single lesion, 2 = multiple lesions, and 3 = diffuse involvement. Crypt abscess formation was evaluated separately and scored on a scale of 0 to 2: 0 = normal, 1 = single lesion, and 2 = multiple lesions. The degree of inflammation was graded on a scale of 0 to 4 based on the depth of inflammatory involvement: 0 = normal; 1 = inflammation limited to the mucosal epithelium and lamina propria; 2 = involvement extending to the muscularis mucosa; 3 = involvement of the submucosa; and 4 = transmural inflammation involving the muscular layer and serosa. The degree of crypt damage was also scored on a scale of 0 to 4: 0 = normal; 1 = damage limited to the basal one-third of the crypt; 2 = damage extending to the basal two-thirds of the crypt; 3 = complete crypt loss; and 4 = crypt destruction accompanied by ulceration. The total epithelial injury score was calculated as the sum of these parameters. The final histological score for each animal was obtained by combining the inflammatory infiltration score and epithelial injury score, with the scoring criteria detailed in Table 1. For each experimental group, multiple sections from at least 4 randomly selected rats were analyzed, and the mean score was calculated to represent the group. In addition, multiple fields per section were examined to ensure representative sampling of the tissue.

**Table 1:** The intestine pathological criteria.

Score	Degree of inflammation	Inflammatory cell infiltration	Degree of damage to the crypts	Crypt abscess	Submucosal edema	Loss of goblet cells	Degree of crypt epithelial hyperplasia
0	Normal	Normal	Normal	Normal	Normal	Normal	Normal
1	Mucosal epithelium + lamina propria	Single lesion	Base 1/3 of crypt	Single lesion	Single lesion	Single lesion	Single lesion
2	Muscularis mucosa	Multiple lesions	Base 2/3 of crypt	Multiple lesions	Multiple lesions	Multiple lesions	Multiple lesions
3	Submucosa	Diffuse	The whole crypt		Diffuse	Diffuse	Diffuse
4	Muscular layer + serosa		Crypt injury + ulcer				

### 2.10 Proinflammatory biomarkers

After the spinal cord tissue samples were collected, they were placed in ice-cold PBS with the addition of a protease inhibitor to prevent protein degradation. Following processing, the samples were centrifuged at 12,000 rpm for 20 min at 4 °C to remove residual debris, and the supernatant was collected. The total protein concentration in the supernatant was determined using the bicinchoninic acid assay (BCA) method to ensure consistent protein amounts across all samples during loading. Quantitative analysis of the TNF-α and IL-1β concentrations in the spinal cord tissue was performed using commercially available enzyme-linked immunosorbent assay (ELISA) kits (TNF-α: FineTest, China, ER1393; IL-1β: FineTest, China, ER1094), strictly following the kit instructions. After the reaction, the optical density (OD) was measured within 30 min using a microplate reader (TeBao, United States) at a wavelength of 450 nm. The measured OD values were compared with the standard curve, which was prepared using known concentrations of TNF-α and IL-1β standards, to calculate the concentrations of these cytokines in the samples. All cytokine concentrations were normalized to the total protein concentration of the sample (in mg), and the final results are expressed in pg./mg. To ensure the reliability and reproducibility of the experimental data, all experiments were performed in triplicate.

### 2.11 Western blot

0.1 g of colon tissue samples were mixed with 1 mL of cold RIPA lysis buffer (Beyotime Biotechnology, Shanghai, China), and then homogenized using a high-speed, low-temperature tissue homogenizer. The homogenization conditions were set at −20 °C, with each round lasting 60 s, repeated four times. After homogenization, the samples were transferred to a 4 °C refrigerator for 30 min of lysis. Subsequently, the samples were centrifuged at 10,000 rpm for 15 min at 4 °C. The supernatant was collected, and protein concentration was measured using a BCA Protein Assay Kit (Beyotime Biotechnology, Shanghai, China). For subsequent analysis, equal amounts of protein were subjected to SDS-PAGE electrophoresis, and the separated proteins were transferred onto a PVDF membrane. After transfer, the membrane was blocked with 5% non-fat milk in TBST buffer for 2 h. The membrane was then incubated overnight with primary antibodies occludin (Rabbit/IgG, Proteintech, 27260-1-AP), claudin-1 (Rabbit/IgG, Proteintech, 28674-1-AP), ZO-1 (Rabbit/IgG, Proteintech, 21773-1-AP) at 4 °C. Following three washes with TBST, the membrane was incubated with the corresponding secondary antibodies at room temperature for 2 h. To ensure consistent sample loading, β-actin was used as the internal control. The grayscale values of the target proteins were quantified using ImageJ software.

### 2.12 Immunofluorescence and quantification of AQP4 polarization

Rats were deeply anesthetized and perfused via the heart with PBS followed by 4% paraformaldehyde (PFA). The L4-L6 spinal segments were rapidly extracted and post-fixed in 4% PFA for 24 h. After fixation, tissues were processed, embedded in paraffin, and sectioned. Paraffin sections were deparaffinized, subjected to antigen retrieval, and treated to block endogenous peroxidase activity and nonspecific binding. Immunofluorescence double labeling was performed to detect AQP4 and CD31 expression. As both primary antibodies were raised in rabbit, a sequential staining protocol with a blocking step was applied to avoid cross-reactivity. Briefly, tissue sections were first incubated with anti-AQP4 antibody (Rabbit/IgG, Proteintech, AB_2827426, 1:100) overnight at 4 °C. After washing three times with PBS (pH 7.4, 5 min each), sections were incubated with a Cy3-conjugated goat anti-rabbit secondary antibody (Servicebio, GB21303) for 1 h at room temperature to visualize AQP4. To prevent cross-reactivity between antibodies from the same host species, sections were subsequently incubated with a purified normal rabbit IgG (A7016, Beyotime, China) for 30 min at room temperature. Following the blocking step, sections were incubated with rabbit anti-CD31 antibody (Rabbit/IgG, Abcam, AB_11218334, 1:100) overnight at 4 °C. After washing, sections were incubated with FITC-conjugated goat anti-rabbit IgG secondary antibody (Proteintech, SA00003-2, 1:200) for 1 h at room temperature. Finally, nuclei were counterstained with DAPI for 10 min, and sections were mounted with antifade mounting medium. Images were acquired using a fluorescence microscope (model device TCS SP5, Leica Microsystems, Germany).

For the quantification of AQP4 expression near blood vessels, images were processed using ImageJ software (version 1.54f, Java 1.8.0_322). We defined Regions of Interest (ROIs) on each image, with clear and consistent criteria for ROI selection applied across all groups. The analysis specifically focused on the gray matter of the spinal cord, with measurements taken from the dorsal horn, ventral horn, and central canal regions. For each spinal cord section, three regions of interest (ROIs) were selected per region. Each ROI was placed to include representative perivascular and parenchymal areas, avoiding overlapping vessels. The AQP4 channel was extracted, background fluorescence was subtracted, and the mean immunofluorescence intensity was measured for each ROI. To assess AQP4 polarization, we adapted the method described in previous study (Kress et al., 2014). AQP4 is predominantly localized to astrocytic end-feet surrounding blood vessels, a distribution referred to as “AQP4 polarization.” To quantify this distribution, double-immunofluorescence images of AQP4 and CD31 were analyzed using ImageJ software (NIH, United States). The AQP4 channel was extracted from merged images, and all images were acquired and processed using identical settings. CD31-positive signals were used to identify vascular structures. For each image, perivascular regions of interest (ROIs) were manually delineated around CD31-positive vessels within the central canal, ventral horn, and dorsal horn regions. The mean AQP4 immunofluorescence intensity within these perivascular ROIs was measured to represent vascular-associated AQP4 expression. A global threshold was then applied to the AQP4 channel and kept constant across all images. Based on this threshold, the percentage of the total image area in which AQP4 fluorescence intensity was lower than the mean perivascular intensity was calculated. This percentage was defined as the “AQP4 polarization.” In this context, a higher polarization index reflects a greater restriction of AQP4 signal to perivascular astrocytic end-feet and reduced expression in the surrounding parenchyma. All measurements were performed in a blinded manner, and multiple sections per animal were analyzed to ensure reproducibility. Based on the above methodology, the AQP4 polarization index can be calculated as follows: AQP4 Polarization Index = (Area with AQP4 fluorescence intensity < Mean perivascular intensity) / (Total image area) × 100. Area with AQP4 fluorescence intensity < Mean perivascular intensity refers to the percentage of the image area where AQP4 fluorescence intensity is lower than the mean intensity measured in the perivascular ROIs. Total image area refers to the full area of the image analyzed.

### 2.13 Analysis of gut microbiota

Following anesthesia, the colon was rapidly incised using a sterile scalpel. Colonic contents were collected and transferred into sterile sampling tubes. Samples were immediately flash-frozen in liquid nitrogen and stored at −80 °C until further analysis. Genomic DNA was extracted using the cetyltrimethylammonium bromide (CTAB) method. Briefly, CTAB lysis buffer and lysozyme were added to a 2.0 mL centrifuge tube containing the sample, followed by incubation in a 65 °C water bath with intermittent inversion to facilitate lysis. DNA was subsequently purified via two rounds of phenol–chloroform extraction, precipitated with isopropanol, and washed with 75% ethanol. The resulting DNA pellet was air-dried under sterile conditions, resuspended in deionized water, and treated with RNase A to remove residual RNA. DNA concentration, integrity, and purity were assessed using the Agilent 5400 system. High-quality DNA samples were used for library construction with the NEBNext® Ultra™ DNA Library Prep Kit for Illumina (NEB, United States). DNA was fragmented to approximately 350 bp using Covaris ultrasonication, followed by end repair, A-tailing, and adapter ligation. Libraries were then amplified by PCR and purified. Library concentration was quantified by qPCR to ensure a final concentration of 1.5 nM, and high-throughput sequencing was performed on the Illumina PE150 platform. Raw sequencing data were preprocessed using KneadData to remove host-derived contamination and low-quality reads. Taxonomic classification was performed using Kraken2 against a custom microbiome database, and sequence counts for bacteria, fungi, archaea, and viruses were obtained. Species-level relative abundances were further estimated using Bracken (Langmead and Salzberg, 2012; Mandal et al., 2015). Functional annotation was conducted using HUMAnN2, with protein database alignment performed via DIAMOND. Functional abundance tables were generated, followed by clustering analyses of species and functional profiles, principal coordinates analysis (PCoA), and non-metric multidimensional scaling (NMDS). Additionally, LEfSe biomarker analysis and Dunn’s test were applied to identify differences in taxonomic and functional composition between groups (Franzosa et al., 2018).

### 2.14 Statistical methods

All data are presented as mean ± standard deviation (SD). Prior to parametric testing, normality was assessed using the Shapiro–Wilk test, and homogeneity of variance was evaluated using Levene’s test. For comparisons between two independent groups with normally distributed data, a two-tailed Student’s *t*-test was applied. For comparisons involving three or more groups, one-way analysis of variance (ANOVA) followed by Tukey’s *post hoc* test was used. Repeated-measures data (e.g., body weight, blood glucose, and PWT) were analyzed using repeated-measures ANOVA, with Geisser–Greenhouse correction applied when the assumption of sphericity was violated. For non-normally distributed data, appropriate non-parametric tests were employed, including the Mann–Whitney U test for two-group comparisons and the Kruskal–Wallis test with Dunn’s *post hoc* correction for multiple comparisons. Gut microbiota analyses were conducted using the bioinformatics platform provided by Shenzhen Microeco Tech Co., Ltd., including α-diversity indices (Shannon and Simpson), β-diversity analysis based on Bray–Curtis distances, and linear discriminant analysis effect size (LEfSe) with false discovery rate correction. All other statistical analyses were performed using GraphPad Prism 8.0, and statistical significance was defined as a two-tailed *p*-value < 0.05.

## 3 Results

### 3.1 GB significantly alleviates mechanical hypersensitivity without affecting hyperglycemia

**Figure 1:**
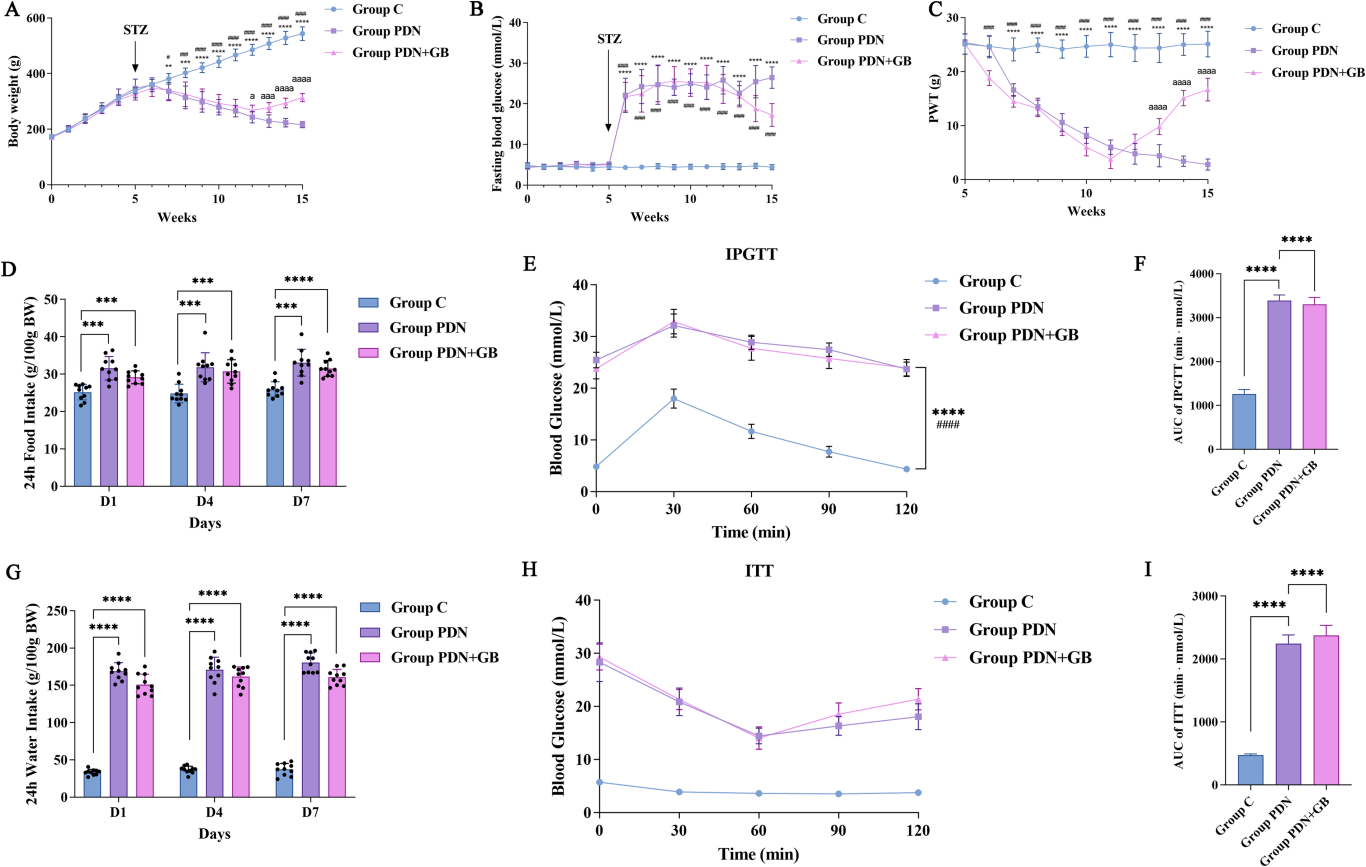
GB effectively alleviates mechanical hypersensitivity without affecting blood glucose levels. **(A)** The trend of body weight in Group C, Group PDN and Group PDN + GB (*n* = 10). **(B)** The trend of blood glucose in Group C, Group PDN and Group PDN + GB (*n* = 10). **(C)** The test of mechanical hypersensitivity from week 5 (streptozotocin injection) to week 15 (*n* = 10). **(D)** The 24-h food intake of rats in the group C, group PDN, and group PDN + GB was recorded on days 1, 4, and 7 of week 6 (*n* = 10); **(E)** Intraperitoneal glucose tolerance test (IPGTT, *n* = 10). **(F)** Area under the IPGTT curve; **(G)** The 24-h water intake of rats in the C, PDN, and PDN + GB groups was also recorded on days 1, 4, and 7 of week 6 (*n* = 10); **(H)** Insulin tolerance test (ITT, *n* = 10). **(I)** Area under the ITT curve. **(A–C)** Group PDN vs. Group C, **p < 0.01, ***p < 0.001, ****p < 0.0001. Group PDN + GB vs. Group C, ^#^*p* < 0.05, ^##^*p* < 0.01, ^###^*p* < 0.001, ^####^*p* < 0.0001. Group PDN + GB vs. Group PDN, ^a^*p* < 0.05, ^aaa^*p* < 0.001, ^aaaa^*p* < 0.0001. **(D–I)** ***p < 0.001, ****p < 0.0001. Group C: Healthy rats; Group PDN: PDN rats; Group PDN + GB: PDN rats were gavaged with Ginkgolide B. Values were presented as mean ± SD.

In this study, body weight in group C increased steadily throughout the experimental period, whereas group PDN and group GB exhibited a significant decline beginning at week 7. Following treatment, body weight in the group PDN + GB began to recover from week 12 (Figure 1A). Regarding fasting blood glucose, group C maintained normoglycemia, while both group PDN and PDN + GB showed a marked increase following streptozotocin (STZ) injection. GB treatment did not significantly ameliorate hyperglycemia (Figure 1B). PWT was assessed weekly from week 5. Compared with group C, PWT values in the group PDN + GB and group PDN decreased significantly at weeks 6 and 7, respectively. After treatment, PWT in the group PDN + GB increased significantly from week 13, indicating that GB effectively alleviated mechanical hypersensitivity in PDN rats (Figure 1C). Food and water intake were monitored over 24-h periods on days 1, 4, and 7 of week 6. Both group PDN and group PDN + GB exhibited marked polyphagia and polydipsia compared with group C (Figures 1D,G). In the intraperitoneal glucose tolerance test (IPGTT) conducted at the beginning of week 6, blood glucose levels in all groups gradually increased following intraperitoneal glucose injection, reaching a peak at 30 min, and then gradually declined. However, compared to the group C, the group PDN and group PDN + GB consistently exhibited higher blood glucose levels throughout the test, with slower recovery to baseline levels (Figure 1E). Additionally, the area under the glucose curve (AUC Glu) in the group PDN and group PDN + GB was significantly higher than that in the group C (****p < 0.0001, Figure 1F), indicating a severe impairment in glucose tolerance in these two groups. In the insulin tolerance test (ITT) conducted at the end of week 6, blood glucose levels gradually decreased after intraperitoneal insulin injection, followed by a slow recovery. However, throughout the testing period, blood glucose levels remained below baseline levels (Figure 1H). Compared to the group C, the AUC Glu values in the group PDN and group PDN + GB were significantly elevated (****p < 0.0001; Figure 1I), suggesting the presence of significant insulin resistance and impaired insulin sensitivity in both groups.

### 3.2 GB reduces spinal cord inflammation and promotes recovery of spinal cord glymphatic system function

**Figure 2:**
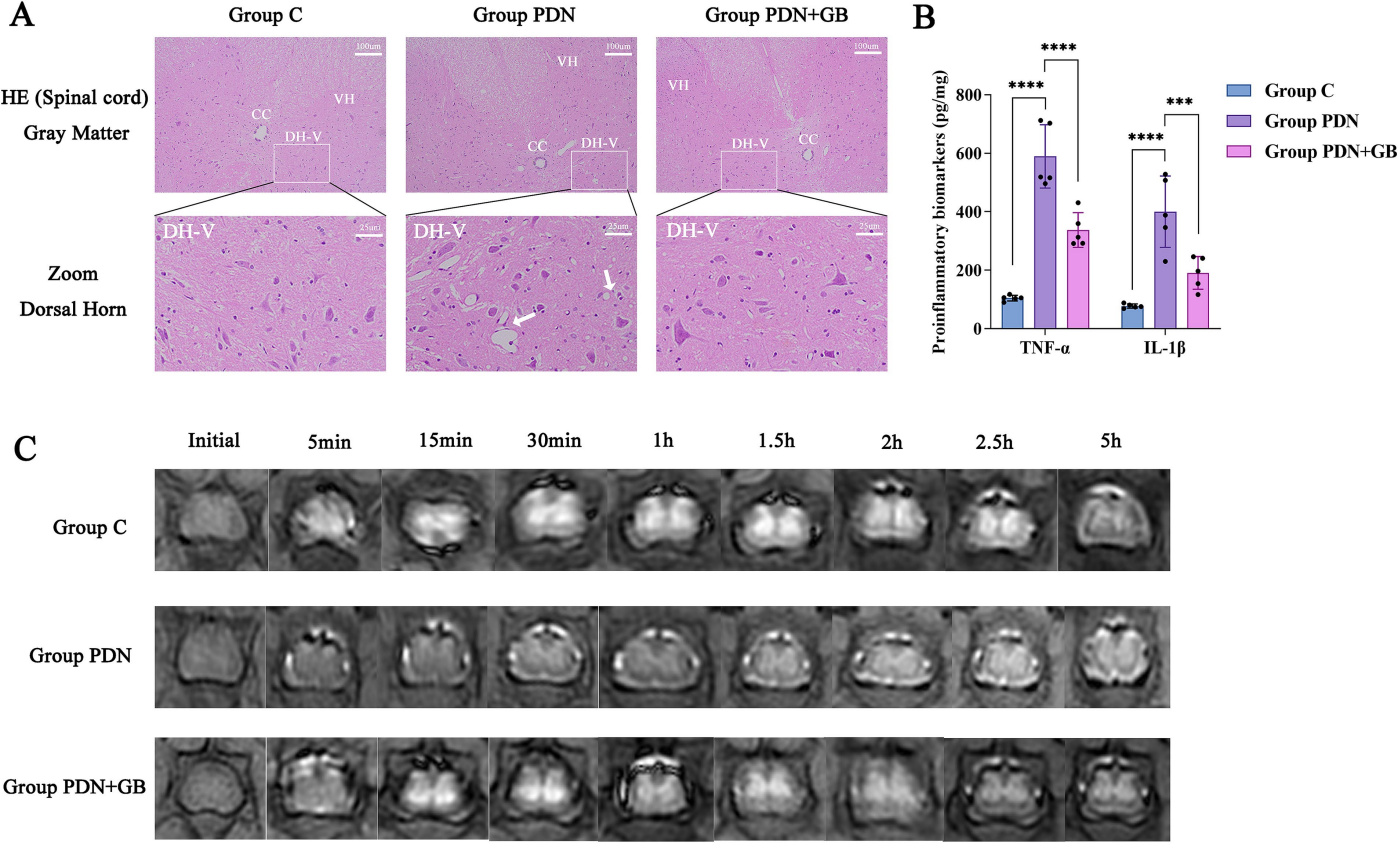
GB reduces spinal cord inflammation and promotes recovery of spinal cord glymphatic system function. **(A)** HE staining results of spinal cord tissue (*n* = 5). Observed under a 20× magnification; Zoom: 80× magnification. Arrows: severe axonal degeneration accompanied by vacuole formation. **(B)** ELISA detection of IL-1 and TNFα levels (*n* = 5). **(C)** Assessment of glymphatic system function in the spinal cord. Initial: Before injection of Gd-DTPA. 5 min: Inject Gd-DTPA for 5 min. 15 min: Inject Gd-DTPA for 15 min. 30 min: Inject Gd-DTPA for 30 min. 1 h: Inject Gd-DTPA for 1 h. 1.5 h: Inject Gd-DTPA for 1.5 h. 2 h: Inject Gd-DTPA for 2 h. 2.5 h: Inject Gd-DTPA for 2.5 h. 5 h: Inject Gd-DTPA for 5 h. ***p < 0.001, ****p < 0.0001. The white box indicates the lamina V region of the dorsal horn. CC, Central Canal; DH-V, dorsal horn, lamina V; VH, Ventral Horn. Refer to Figure 1 for the meaning of the grouping abbreviation. Values were presented as mean ± SD.

In the HE-stained spinal cord tissue images, group PDN exhibited significant vacuolization of neurons in the gray matter, along with severe axonal degeneration and vacuole formation (indicated by arrows), compared to group C. Additionally, noticeable infiltration of inflammatory cells was observed in the group PDN. However, treatment with GB led to substantial improvement in these pathological changes (Figure 2A). ELISA analysis of spinal cord tissue revealed significantly elevated levels of inflammatory cytokines IL-1β and TNF-α in group PDN compared to group C (IL-1β: group PDN 400.2 ± 105.51 vs. group C 77.71 ± 7.33, ****p < 0.0001; TNF-α: group PDN 589.4 ± 92.06 vs. group C 104.8 ± 9.15, ****p < 0.0001). Following GB treatment, the levels of IL-1β (group PDN + GB 191.2 ± 49.32) and TNF-α (group PDN + GB 337.6 ± 54.60) were significantly reduced (Figure 2B). These results indicate that GB effectively attenuates the inflammatory response in the spinal cord, thereby mitigating inflammation-induced damage.

To further evaluate the function of the spinal cord glymphatic system, MRI was employed to monitor the distribution and metabolic dynamics of the contrast agent within the spinal cord. As shown in Figure 2C, the contrast agent gradually accumulated in the gray matter, forming a characteristic “butterfly sign” pattern. Compared with the group C and group PDN + GB, the group PDN exhibited a delayed appearance of the “butterfly sign,” suggesting altered dynamics of contrast agent distribution in the spinal cord. To quantitatively assess these changes, MRI signal intensities in the gray matter regions were analyzed to determine the absorption and clearance rates of the contrast agent. The results demonstrated that both the absorption rate (Group C: 1877 ± 268.45, Group PDN: 974 ± 152.17, Group PDN + GB: 1730 ± 242.65) and the clearance rate (Group C: 747 ± 83.44, Group PDN: 308 ± 35.72, Group PDN + GB: 547 ± 100.65) were significantly reduced in the group PDN compared with the group C and group PDN + GB (Table 2). These quantitative findings indicate a slower influx and efflux of the contrast agent in the group PDN, which may reflect impaired glymphatic transport function in the spinal cord. Notably, treatment with GB partially restored these parameters, suggesting an improvement in spinal cord metabolic and clearance capacity.

**Table 2:** Quantitative analysis of the absorption and clearance rates of the contrast agent by the spinal cord glymphatic system.

Parameters	Group C (*n* = 3)	Group PDN (*n* = 3)	Group PDN + GB (*n* = 3)
Initial SI	6,154 ± 122.74	6,697 ± 53.92	6,769 ± 98.76
Peak SI	9,224 ± 87.39	8,774 ± 187.69	9,364 ± 279.82
5 h SI	6,753 ± 118.94	7,901 ± 70.28	7,450 ± 261.02
T1	1.67 ± 0.24	2.17 ± 0.24	1.5
V1	1,877 ± 268.45*	974 ± 152.17	1,730 ± 242.65*
T2	3.33 ± 0.24	2.83 ± 0.24	3.5
V2	747 ± 83.44**	308 ± 35.72	547 ± 100.65*

Initial SI: the initial SI. Peak SI: the peak of SI. 5 h SI: SI values 5 h post-injection. T1: the time to Peak SI. VI: the rate of change in SI, representing the rate at which the signal intensity increases from Initial SI to Peak SI. The calculation formula is V1 = (Mean(Peak SI)-Mean(Initial SI))/Mean(T1). T2: 5-T1. V2: the rate of change in SI, representing the rate at which the signal intensity decreases from Peak SI to 5 h SI. The calculation formula is V2 = (Mean(PEAK SI)-Mean(5 h SI))/Mean(T2). Compared with group PDN, *p < 0.05, **p < 0.01. Refer to Figure 1 for the meaning of the grouping abbreviation. Values were presented as mean ± SD.

### 3.3 Regulation of AQP4 localization and expression in the spinal cord glymphatic system by GB

**Figure 3:**
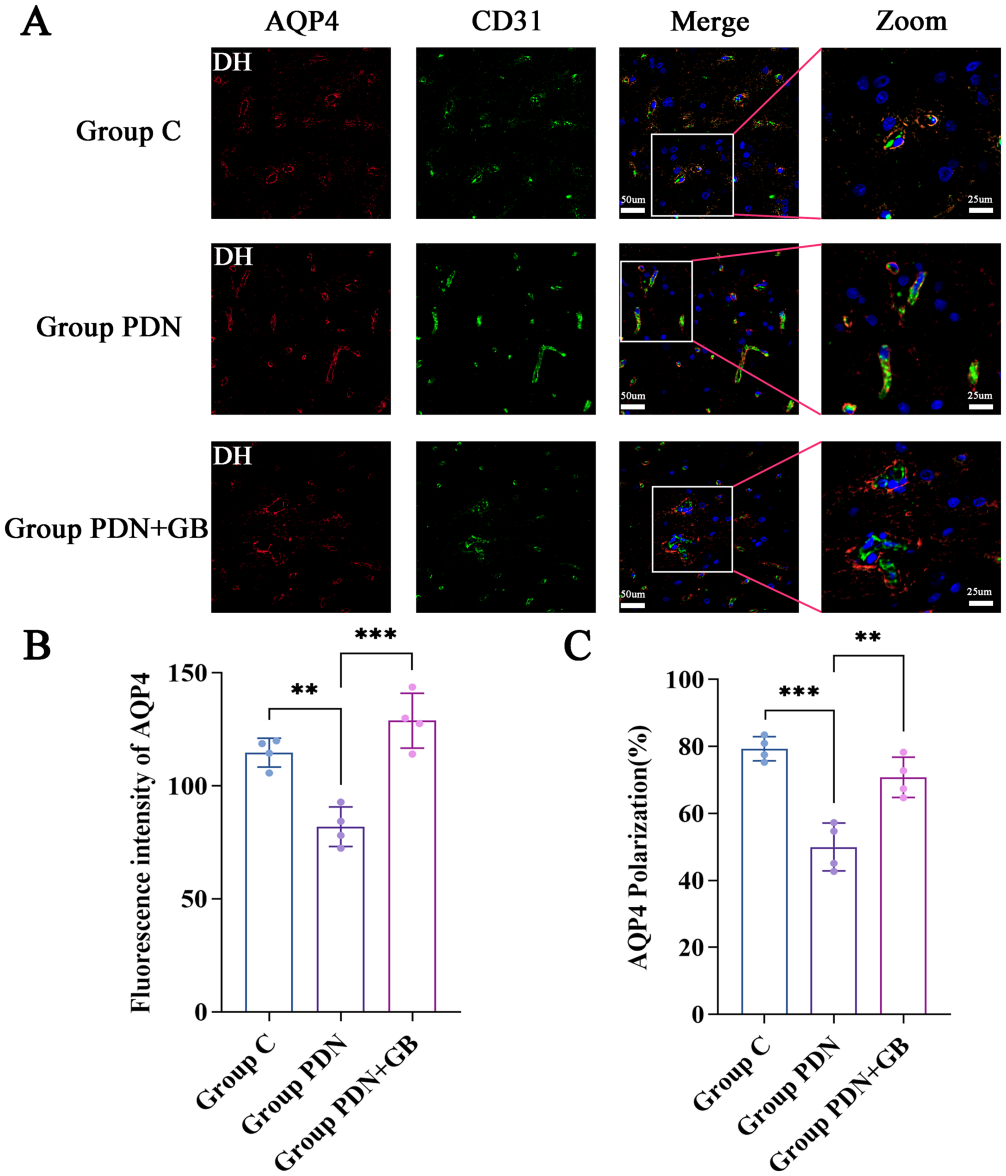
Immunofluorescence results of AQP4 and CD31. **(A)** Representative images of AQP4 and CD31 staining in the spinal cord (*n* = 4). Merge: 20× magnification, zoom: 40× magnification, green fluorescence: CD31 (Blood vessels), red fluorescence: AQP4, blue fluorescence: cell nuclei were counterstained with DAPI. Enlarged sections detailed the polarity reversal of AQP4 protein. **(B)** Quantitative analysis of fluorescence in AQP4 across the groups (*n* = 4). **(C)** Quantitative analysis of fluorescence polarization of AQP4 (*n* = 4). DH: Dorsal horn of the spinal cord. **p < 0.01, ***p < 0.001. Refer to Figure 1 for the meaning of the grouping abbreviation. Values were presented as mean ± SD.

AQP4, a key structural component of the spinal cord glymphatic system, was evaluated for its expression and localization. To assess these parameters, the endothelial cell-specific marker CD31 (green fluorescence) was used to label blood vessels. In group C, AQP4 exhibited strong fluorescence surrounding the blood vessels, with a continuous pattern consistent with the previously described “polarized distribution.” In contrast, group PDN showed disrupted AQP4 fluorescence around the vasculature, accompanied by a marked reduction in fluorescence intensity. Following GB treatment, AQP4 fluorescence intensity was enhanced, and the continuity of its perivascular distribution was largely restored compared to group PDN (Figure 3A). These findings indicate that the polarized distribution of AQP4 in the spinal cord glymphatic system is impaired in PDN rats, whereas GB treatment partially restores this distribution. Quantitative analysis further demonstrated that AQP4 fluorescence intensity was significantly reduced in group PDN compared with groups C and PDN + GB (group C: 114.7 ± 5.48; group PDN: 81.92 ± 7.01; group PDN + GB: 128.8 ± 11.94), suggesting a substantial decrease in AQP4 expression in PDN rats that was partially reversed by GB treatment (Figure 3B). Additionally, ImageJ-based quantification of perivascular AQP4 distribution revealed a significant reduction in the polarization ratio in group PDN compared with groups C and PDN + GB (group C: 79.32% ± 3.02%; group PDN: 49.97% ± 6.41%; group PDN + GB: 70.8% ± 5.84%; Figure 3C). These results further confirm the disruption of AQP4 polarization in PDN rats and demonstrate that GB treatment effectively ameliorates this impairment.

### 3.4 GB promotes intestinal barrier repair

**Figure 4:**
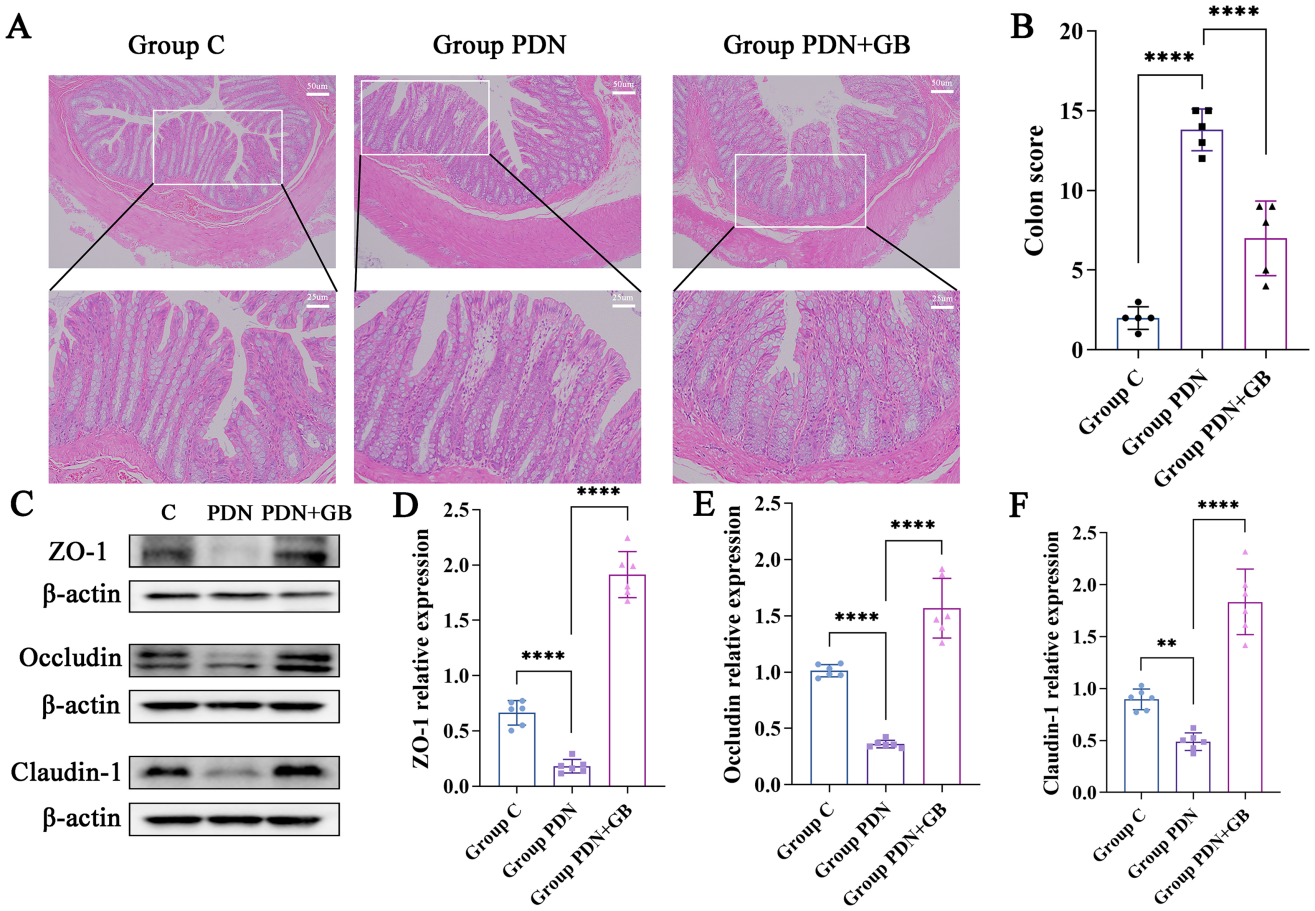
GB promotes intestinal barrier repair. **(A)** HE staining results of colonic tissue (*n* = 5). Observed under a 20× magnification. **(B)** Colonic tissue pathological scoring (*n* = 5). **(C)** Expression levels of intestinal barrier-related proteins. **(D)** Relative expression of ZO-1 (*n* = 5). **(E)** Relative expression of occludin (*n* = 5). **(F)** Relative expression of claudin (*n* = 5). Compared with group PDN, **p < 0.01, ****p < 0.0001. Refer to Figure 1 for the meaning of the grouping abbreviation. Values were presented as mean ± SD.

Histopathological analysis of H&E-stained colon sections revealed that, compared with group C and group PDN + GB, colonic tissue in group PDN exhibited pronounced inflammatory infiltration and extensive villous damage (Figure 4A), indicating more severe inflammatory injury in PDN rats. To quantitatively assess colonic tissue damage, pathological scoring was performed according to the criteria in Table 1. As shown in Figure 4B, the pathological score of group PDN was significantly higher than that of group C (group C: 2 ± 0.71; group PDN: 13.8 ± 1.30; *P* < 0.0001), further confirming the severity of colonic injury in PDN rats. GB treatment markedly reduced the pathological score (group PDN + GB: 7 ± 2.16), suggesting its potential in alleviating intestinal inflammatory damage. ZO-1, Occludin, and Claudin-1 are critical components of intestinal tight junctions and are essential for maintaining the structure and function of intestinal epithelial cells. Figures 4C–F illustrate the expression levels of these proteins. In group PDN, the expression of ZO-1, Occludin, and Claudin-1 was significantly decreased compared with group C (ZO-1: 0.18 ± 0.06 vs. 0.66 ± 0.11, ****P < 0.0001; Occludin: 0.36 ± 0.04 vs. 1.01 ± 0.05, ****P < 0.0001; Claudin-1: 0.47 ± 0.09 vs. 0.90 ± 0.01, **P < 0.01). Notably, GB treatment restored the expression of these transmembrane proteins to levels exceeding those in group C (ZO-1: 1.91 ± 0.23; Occludin: 1.57 ± 0.25; Claudin-1: 1.75 ± 0.33), indicating that GB may enhance intestinal barrier protein expression and facilitate repair of the damaged intestinal mucosa.

### 3.5 The modulatory effects of GB on mechanical hypersensitivity and AQP4 are dependent on the gut microbiota

**Figure 5:**
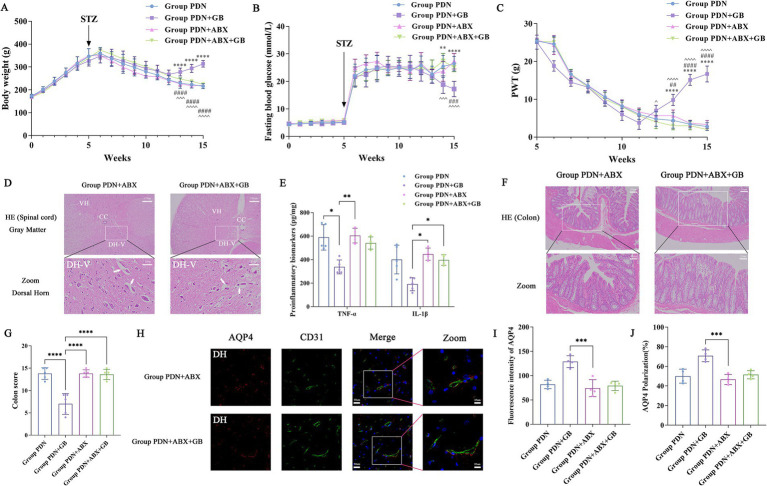
The modulatory effects of GB on mechanical hypersensitivity and AQP4 are dependent on the gut microbiota. **(A)** The trend of body weight in Group PDN, Group PDN + GB, Group PDN + ABX and Group PDN + ABX + GB (*n* = 6); **(B)** The trend of fasting blood glucose in Group PDN, Group PDN + GB, Group PDN + ABX and Group PDN + ABX + GB (*n* = 6); **(C)** The test of mechanical hypersensitivity from week 5 (streptozotocin injection) to week 15 (*n* = 6); **(D)** HE staining results of spinal cord tissue (*n* = 3). Observed under a 20× magnification; Zoom: 80× magnification. Arrows: severe axonal degeneration accompanied by vacuole formation; **(E)** ELISA detection of IL-1 and TNFα levels (*n* = 4); **(F)** HE staining results of colonic tissue (*n* = 3); **(G)** Colonic tissue pathological scoring (*n* = 5); **(H)** Representative images of AQP4 and CD31 staining in the spinal cord (*n* = 4). Green fluorescence: CD31 (Blood vessels), red fluorescence: AQP4, blue fluorescence: cell nuclei were counterstained with DAPI. Enlarged sections detailed the polarity reversal of AQP4 protein; **(I)** Quantitative analysis of fluorescence in AQP4 across the groups (*n* = 4); **(J)** Quantitative analysis of fluorescence polarization of AQP4 (*n* = 4). The white box indicates the lamina V region of the dorsal horn. CC, Central Canal; DH-V, dorsal horn, lamina V; VH, Ventral Horn. *P < 0.05, **P < 0.01, ***P < 0.001, ****P < 0.0001. Refer to Figure 1 for the meaning of the grouping abbreviation. Values were presented as mean ± SD.

To clarify whether the gut microbiota contributes to the effects of GB on mechanical hypersensitivity and spinal glymphatic function, PDN rats were treated with a broad-spectrum antibiotic cocktail to reduce microbial diversity and abundance. After antibiotic administration, body weight changes in the group PDN + ABX + GB and group PDN + ABX were comparable to those in the group PDN, and blood glucose levels remained consistently elevated across all groups (Figures 5A,B). From week 14 onwards, the PWT was markedly lower in the group PDN, group PDN + ABX + GB, and group PDN + ABX than in the group PDN + GB (Figure 5C), suggesting that depletion of the gut microbiota weakened the analgesic effect of GB. Histological examination of the spinal cord further revealed evident neuronal damage in both the group PDN + ABX + GB and group PDN + ABX (Figure 5D). In parallel, the expression of pro-inflammatory cytokines, including IL-1β and TNF-α, was significantly increased in these groups compared with the group PDN + GB (Figure 5E; TNF-α: group PDN: 589.4 ± 92.06; group PDN + GB: 337.6 ± 54.60; group PDN + ABX: 604.4 ± 61.9; group PDN + ABX + GB: 541.1 ± 45.6; IL-1β: group PDN: 400.2 ± 105.5; group PDN + GB: 191.2 ± 49.32; group PDN + ABX: 446.3 ± 43.71; group PDN + ABX + GB: 396.3 ± 35.58). In the colon, both the group PDN + ABX and group PDN + ABX + GB showed pronounced inflammatory cell infiltration and disruption of villus architecture (Figure 5F), accompanied by higher pathology scores than those in the group PDN + GB (Figure 5G; group PDN: 13.8 ± 1.3; group PDN + GB: 7 ± 2.16; group PDN + ABX: 13.8 ± 0.84; group PDN + ABX + GB: 13.6 ± 1.14). These findings indicate that antibiotic-induced disruption of the gut microbiota diminishes, and may even negate, the anti-inflammatory and barrier-protective effects of GB. Further immunofluorescence analysis of spinal cord tissues showed a clear reduction in AQP4 fluorescence intensity in the group PDN + ABX and group PDN + ABX + GB. In addition, the polarized distribution of AQP4 was disrupted, displaying a discontinuous pattern (Figure 5H). Quantitative analysis confirmed that both AQP4 intensity and polarization were significantly decreased in the group PDN, group PDN + ABX + GB, and group PDN + ABX compared with the group PDN + GB (Figures 5I,J; AQP4 intensity: group PDN: 81.92 ± 7.01; group PDN + GB: 128.8 ± 11.94; group PDN + ABX: 74.21 ± 18.13; group PDN + ABX + GB: 79.06 ± 9.48; AQP4 polarization: group PDN: 49.97 ± 6.41%; group PDN + GB: 70.8 ± 5.84%; group PDN + ABX: 46.63% ± 5.18%; group PDN + ABX + GB: 51.45% ± 4.47%), indicating that antibiotic treatment impairs the regulatory effect of GB on AQP4. Taken together, these results suggest that GB may exert its anti-inflammatory and analgesic effects, as well as its roles in intestinal barrier repair, spinal glymphatic function, and AQP4 organization, at least in part through modulation of the gut microbiota.

### 3.6 The impact of GB on the gut microbiota

#### 3.6.1 Effect of GB on α diversity and β diversity of gut microbiota

**Figure 6:**
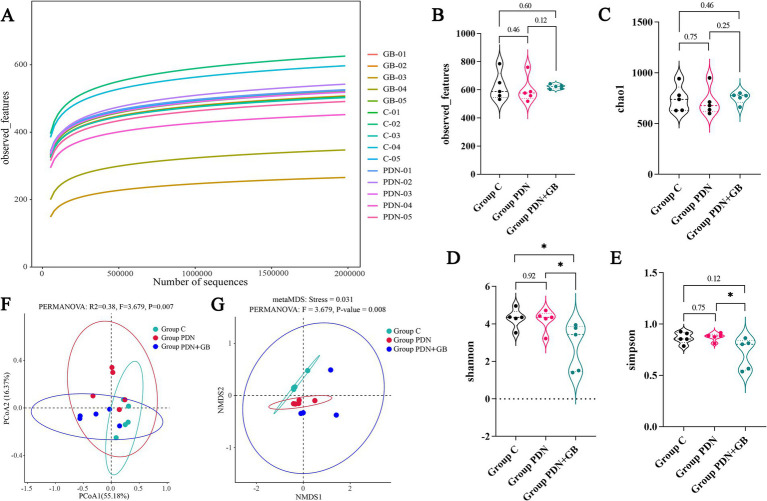
Effect of GB on α diversity and β diversity of gut microbiota. **(A)** The rarefaction curve (*n* = 5 rats/group). The abscissa is the number of sampling sequences and the ordinate is the number of observed features in the sampling sequences. **(B)** Observed features. **(C)** Chao1 index. **(D)** Shannon index. **(E)** Simpson index. **(F)** Principal Component Analysis (PCA) of gut microbiota. **(G)** Non-metric multidimensional scaling of gut microbiota. *N* = 5 rats/group, Kruskal-Wallis H followed by Tukey’s *post hoc* test performed to evaluate statistical significance. *p < 0.05. Refer to Figure 1 for the meaning of the grouping abbreviation. Values were presented as mean ± SD.

Alpha (α) and beta (β) diversity are key indices for assessing microbial community diversity, each reflecting distinct aspects of community structure. As shown in Figure 6A, the observed-feature curve gradually plateaued when the sequencing depth exceeded 200,000 reads, and no substantial increase in observed features was detected with further sequencing. This indicates that the sequencing depth reached saturation, capturing the majority of microbial species and adequately reflecting gut microbiota diversity. Figures 6B–E present α diversity metrics of the rat gut microbiota across the three groups, including observed features, Shannon, Simpson, and Chao1 indices. No statistically significant differences were observed among the groups, suggesting comparable species richness and evenness. In contrast, β diversity analysis based on non-metric multidimensional scaling (NMDS) and principal coordinates analysis (PCoA) (Figures 6F,G) revealed significant differences among the groups, indicating distinct microbial community compositions. These findings suggest that, although α diversity remained similar, β diversity analysis detected substantial alterations in community structure, potentially attributable to differences in treatment conditions.

#### 3.6.2 Effects of GB on the composition and abundance of the gut microbiota at the phylum and genus levels

**Figure 7:**
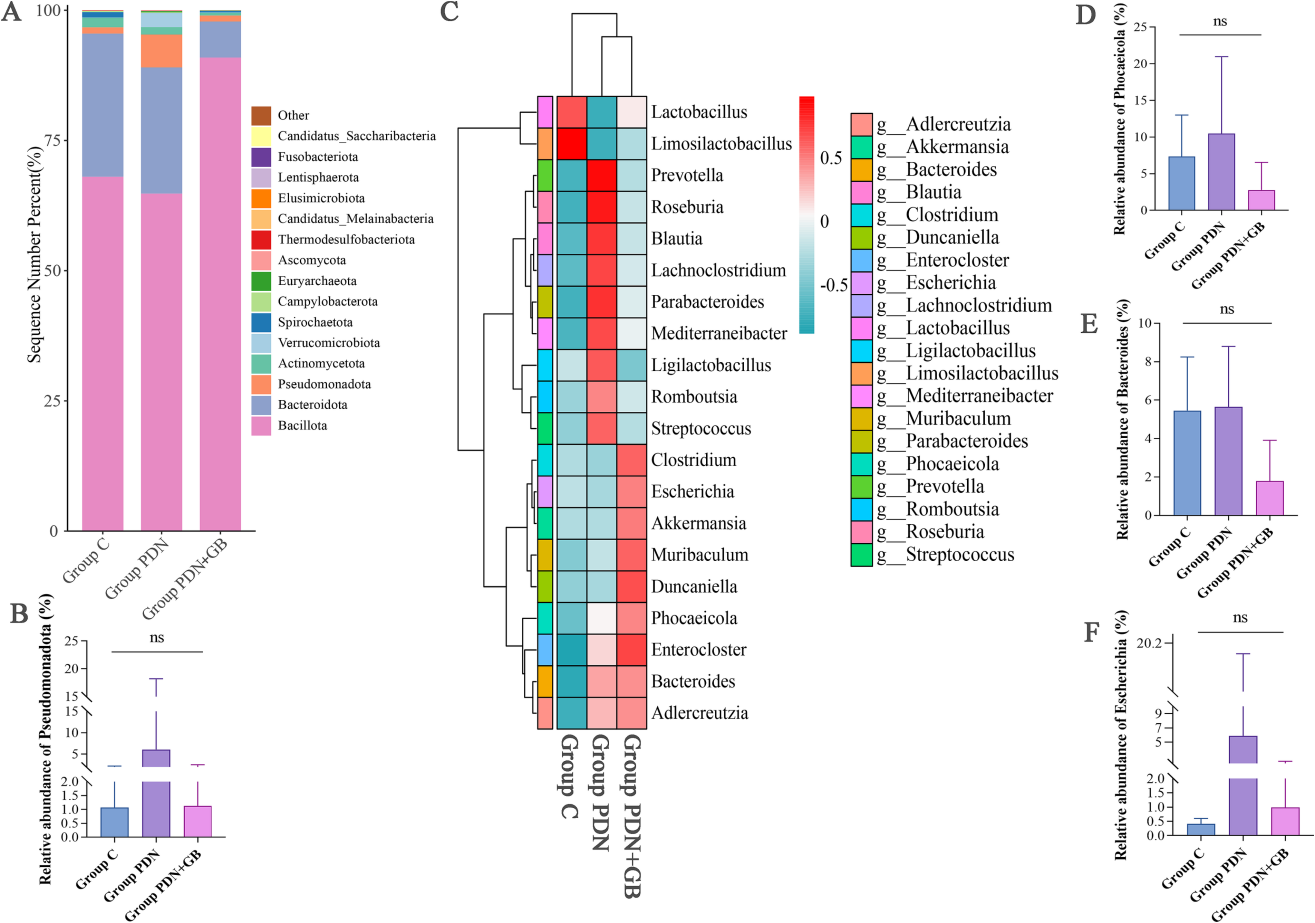
Effects of GB on the composition and abundance of the gut microbiota at the phylum and genus levels. **(A)** Relative abundance at the phylum level. **(B)** Relative abundance of the Pseudomonadota. **(C)** Heatmap of clustering analysis at the genus level. **(D)** Relative abundance of the *Phocaeicola*. **(E)** Relative abundance of the *Bacteroides*. **(F)** Relative abundance of the *Escherichia*. *n* = 5 rats/group, Kruskal–Wallis H followed by Tukey’s *post hoc* tests statistics of the relative **(B)** bacterial phyla and **(D–F)** bacterial genus abundances in group C, group PDN and group PDN + GB. Refer to Figure 1 for the meaning of the grouping abbreviation. Values were presented as mean ± SD.

To investigate the impact of GB on gut microbiota structure, we analyzed the relative abundance of microbial taxa at the phylum level across the three groups. The results showed that, compared with group C and group PDN, the group PDN + GB exhibited a significantly higher abundance of *Bacillota* and a significantly lower abundance of *Bacteroidota* (Figure 7A). Consequently, the *Bacillota*/*Bacteroidota* ratio was elevated in the group PDN + GB, which may represent a distinctive feature of its gut microbiota composition and could be associated with its therapeutic effects or other pharmacological properties. Additionally, the relative abundance of *Pseudomonadota* was higher in the group PDN than in group C and group PDN + GB (Figure 7B), suggesting a potential enrichment of this phylum in PDN and its possible involvement in disease progression. At the genus level, we further examined microbial composition. Figure 7C shows the top 20 bacterial genera in each group, where intergroup differences were more pronounced, indicating distinct microbial profiles. Subsequent statistical analyses revealed that the genera *Phocaeicola*, *Bacteroides*, and *Escherichia* were significantly more abundant in the group PDN than in the group C, with a decreasing trend following GB treatment (Figures 7D–F). *Phocaeicola* and *Bacteroides* are known to participate in the degradation of complex carbohydrates and dietary fibers, contributing to gut homeostasis, immune regulation, and metabolic processes. *Escherichia* is associated with intestinal inflammation and immune responses. Therefore, alterations in the abundance of these genera may reflect the pathophysiological mechanisms underlying diabetic neuropathy and highlight potential targets for gut microbiota–based therapeutic interventions.

#### 3.6.3 Bacterial taxa differences among different groups and redundancy analysis

**Figure 8:**
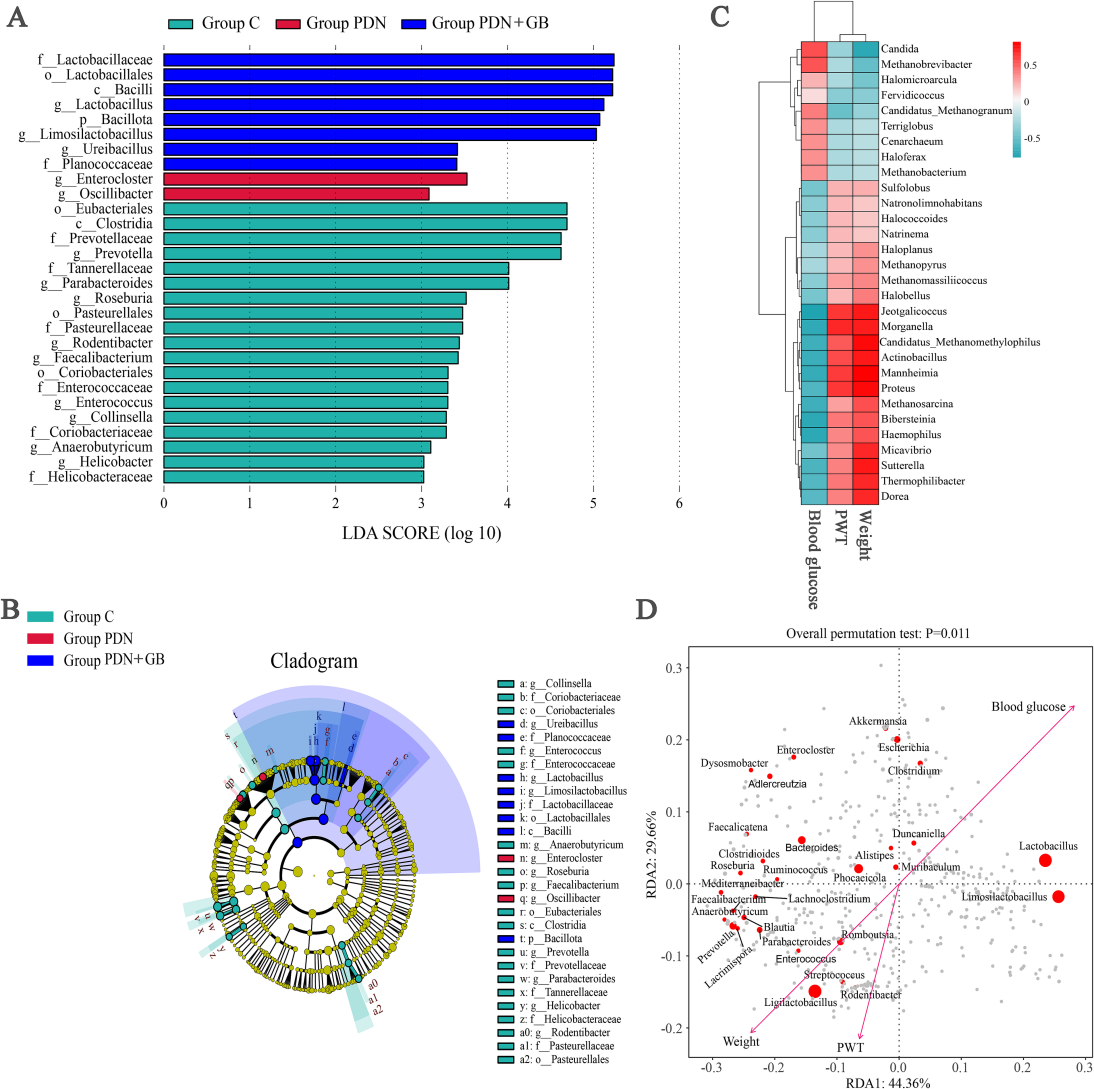
Bacterial taxa differences among different groups and redundancy analysis. **(A)** LEfSe analysis was performed to identify the bacterial taxa differentially represented in group C, group PDN and group PDN + GB at different taxonomy levels (LDA = 3). **(B)** Cladogram generated by the LEfSe analysis showing enriched taxa in feces from the group PDN + GB, group PDN and group C. The circle radiating from inside to outside represents the classification level from phyla to genus, and the diameter represents the size of relative abundance. **(C)** Heatmap of correlations between gut microbiota abundance and weight, blood glucose and PWT. The intensity of the color represents the *r*-value of Spearman’s correlations between the relative abundance of the species and weight, blood glucose and PWT. (negative score, blue; positive score, red). **(D)** Redundancy analysis. Arrows (environmental factors), points (bacteria). LEfSe, Linear discriminant analysis coupled with effect size; LDA, linear discriminant analysis; *n* = 5/group. Refer to Figure 1 for the meaning of the grouping abbreviation.

To characterize the gut microbiota across different rat groups, we performed LEfSe (Linear Discriminant Analysis Effect Size) to identify taxa with differential abundance. The linear discriminant analysis (LDA) effect size threshold was set to 3. It should be noted that the LDA score reflects the magnitude of group separation (effect size), rather than statistical significance per se. A higher LDA score indicates a stronger contribution of a given taxon to the differentiation between groups. The results at multiple taxonomic levels (from phylum to genus) are shown in Figures 8A,B. At the genus level, *g_Enterocloster* and *g_Oscillibacter* were enriched in the group PDN, whereas *g_Lactobacillus*, *g_Limosilactobacillus*, *g_Roseburia*, and *g_Faecalibacterium* were more abundant in the group PDN + GB.

To further investigate associations between gut microbiota and host phenotypes, correlation analysis was conducted between 30 microbial genera and physiological indicators, including body weight, blood glucose, and PWT. As shown in Figure 8C, several genera exhibited significant correlations with these indicators. Specifically, Sulfolobus, Proteus, Morganella, and Dorea were positively correlated with body weight and PWT, but negatively correlated with blood glucose. In contrast, Candida and Methanobrevibacter showed negative correlations with body weight and PWT, and positive correlations with blood glucose. Redundancy analysis (RDA) was performed to assess the influence of environmental factors on microbial community composition, using a correlation coefficient threshold of 0.4. The results indicated that body weight, PWT, and blood glucose significantly contributed to the variation in gut microbiota structure (*p* = 0.011; Figure 8D). In addition, *Enterocloster* and *Clostridium* were positively associated with body weight and PWT, whereas *Lactobacillus* and *Dysosmobacter* showed negative associations with these indicators. Furthermore, *Lactobacillus* and *Limosilactobacillus* were negatively correlated with blood glucose.

#### 3.6.4 Analysis of relative abundance and differences in the biological functions of rat gut microbiota

**Figure 9:**
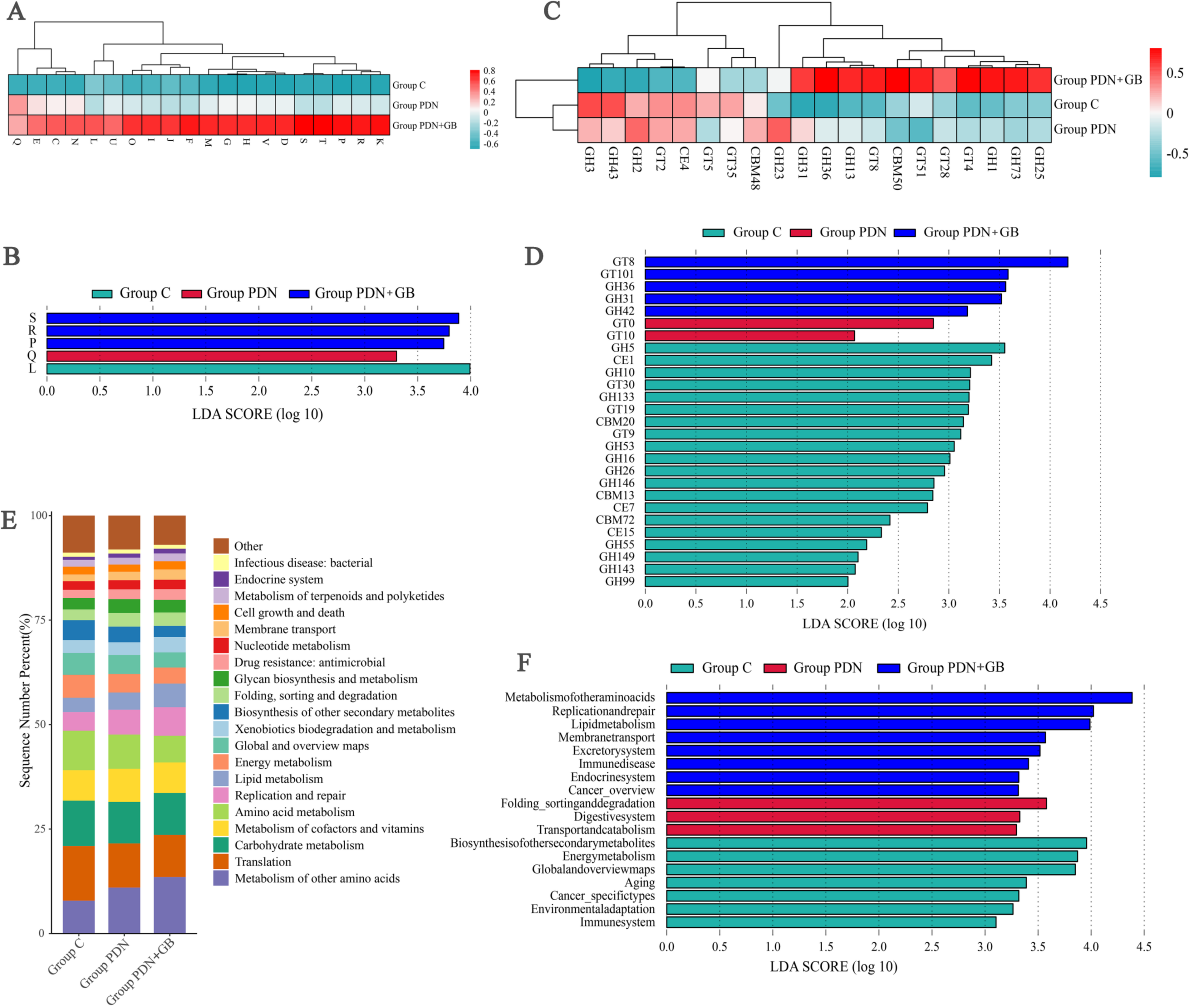
Analysis of relative abundance and differences in the biological functions of rat gut microbiota. **(A)** Heat map of relative abundance of functional annotations in EggNOG. J: Translation, ribosomal structure and biogenesis; R: General function prediction only; L: Replication, recombination and repair; G: Carbohydrate transport and metabolism; S: Function unknown; E: Amino acid transport and metabolism; K: Transcription; F: Nucleotide transport and metabolism; C: Energy production and conversion; M: Cell wall/membrane/envelope biogenesis; P: Inorganic ion transport and metabolism; Q: Secondary metabolites biosynthesis, transport and catabolism. **(B)** Histogram of the distribution of LDA values for differing biological functions of rat gut microbiota (EggNOG). P: Inorganic ion transport and metabolism; Q: Secondary metabolites biosynthesis, transport and catabolism. **(C)** Functional abundance clustering heat map in CAZy. GH, Glycoside Hydrolases; GT, Glycosyl Transferases; CBM, Carbohydrate-Binding Modules. **(D)** Histogram of the distribution of LDA values for differing biological functions of rat gut microbiota (EggNOG). CE, Carbohydrate Esterases. **(E)** Histogram of relative abundance of functional annotations in KEGG (level 2). **(F)** Histogram of the distribution of LDA values for differing biological functions of rat gut microbiota (KEGG, level2). Refer to Figure 1 for the meaning of the grouping abbreviation.

Functional statistical analysis based on the EggNOG database revealed that, compared to group C, the group PDN exhibited significant reductions in core metabolic functions, such as carbohydrate metabolism (G) and energy conversion (E), likely reflecting metabolic dysregulation of the gut microbiota. Additionally, a marked decrease in functions related to cell wall/membrane synthesis (M) suggests potential damage to the intestinal barrier and an underlying inflammatory response. After GB treatment, the functions of carbohydrate metabolism (G), energy conversion (E), and cell wall/membrane synthesis (M) partially recovered, approaching the levels seen in group C (Figure 9A). These results indicate that PDN rats experience functional dysbiosis of the gut microbiota, characterized by diminished metabolic capacity. Notably, GB treatment partially restored these microbial functions, suggesting that GB may exert protective effects by modulating gut microbial metabolism and maintaining structural integrity. LEfSe analysis further revealed significant functional differences in the gut microbiota among the three groups, predominantly focusing on metabolic pathways (P, Q) and genetic information processing (L), suggesting that the gut microbiota responds to both disease status and therapeutic interventions through multiple pathways (Figure 9B).

Analysis using the CAZy database indicated that, compared to group C, the PDN group showed a significant increase in the abundance of enzyme families involved in complex carbohydrate metabolism, such as GH23 and CBM48, while the abundance of enzyme families linked to carbohydrate synthesis, such as GT5 and GT35, was significantly reduced. This suggests that the gut microbiota in the group PDN is impaired in carbohydrate metabolism, which may exacerbate the dysbiosis of the intestinal microbiota. Following GB treatment, the abundance of these key metabolic enzymes was partially restored, indicating that GB may alleviate microbiota imbalance induced by diabetic neuropathy by modulating carbohydrate metabolism (Figure 9C). Furthermore, the LDA score distribution map from LEfSe analysis revealed significant differences in CAZy functional modules among the three groups (Figure 9D). In group C, enzyme families such as GT8 and GT101, associated with glycosyltransferase activities, were significantly enriched, suggesting that a healthy gut microbiota relies on specific glycan synthesis pathways. In contrast, the group PDN showed significant upregulation of glycoside hydrolase families (e.g., GH36, GH31) and cellulose-binding modules (e.g., CBM20), which may be related to an enhanced capacity for complex polysaccharide degradation in the hyperglycemic environment. The group PDN + GB was predominantly enriched in GH146, CBM13, and esterases such as CE7, suggesting that GB treatment may partially reverse the abnormal metabolic features of the group PDN by regulating esterases and fiber-binding functions.

Functional annotation of the gut microbiota metagenomes from the three groups was performed using the KEGG database, followed by LEfSe analysis to identify intergroup differences. The results showed that the gut microbiota functions in all three groups were primarily centered around carbohydrate metabolism, amino acid metabolism, and energy metabolism. Additionally, pathways related to cofactor and vitamin metabolism, as well as lipid metabolism, were also highly abundant, indicating that the gut microbiota plays a crucial role in maintaining the host’s nutritional and metabolic stability, which is fundamental to the development and treatment of PDN (Figure 9E). Further analysis revealed that group C had higher LDA scores (log10 LDA > 3.5) in basic metabolic pathways such as amino acid metabolism, membrane transport systems, and protein folding/sorting and degradation, suggesting that its gut microbiota metabolism is in a balanced homeostatic state. In contrast, the group PDN exhibited significant enrichment in immune disease-related pathways and cancer-specific pathways, along with suppression of lipid metabolism and energy metabolism pathways. This may be closely associated with the chronic inflammation and metabolic dysregulation observed in diabetic neuropathy. Notably, the group PDN + GB showed a significant reversal of the abnormal activation of immune disease-related pathways and restored the biosynthesis of other secondary metabolites. Furthermore, the group PDN + GB exhibited high enrichment in pathways related to environmental adaptation and global metabolic networks, suggesting that GB treatment may enhance the overall coordination of the gut microbiota’s metabolic network, thereby exerting therapeutic effects (Figure 9F).

## 4 Discussion

This study demonstrates that the PDN rat model exhibits characteristic pathological alterations at multiple levels, including peripheral neuropathic pain, spinal cord inflammation, dysfunction of the spinal glymphatic system, and disturbances in both the composition and function of the gut microbiota. GB effectively alleviates these pathological changes, primarily through remodeling of the gut microbiota, promotion of intestinal barrier repair, and attenuation of inflammatory responses in both the intestine and spinal cord, thereby improving PDN symptoms. Furthermore, GB may facilitate the restoration of spinal glymphatic function by regulating the expression and polarization of AQP4, thereby enhancing the clearance of inflammatory mediators from the spinal cord and further mitigating neuroinflammation. Collectively, these findings elucidate potential mechanisms by which GB modulates the microbiome–gut–spinal axis to ameliorate PDN pathology.

In recent years, the interaction between that, in the absence of gut microbiota, PDN mice develop thermal hyperalgesia, mechanical hypersensitivity, and activation of spinal glial cells; however, these pathological manifestations can be markedly reduced or completely prevented under microbiota-deficient conditions. Further studies have identified specific bacterial taxa, including *Akkermansia*, *Bacteroides*, and members of *Desulfovibrionaceae*, as key contributors to this process (Ma et al., 2022). In addition, a two-sample Mendelian randomization study demonstrated that *Prevotella 9* and *Bacteroides* may exert protective effects by slowing PDN progression, whereas *Ruminococcus 2* is positively associated with PDN onset (Xu et al., 2024). Another study found that peripheral neuropathy induced by a high-fat diet is closely related to changes in the gut microbiota, particularly microorganisms such as *Lactobacillus*, *Lachnoclostridium*, and *Anaerotruncus*, which are significantly associated with the transcriptome of the sciatic nerve and involve inflammation, lipid metabolism, and antioxidant defense, all of which are known mechanisms of peripheral neuropathy (Guo et al., 2023). Further research has shown that abnormal changes in the gut microbiota, particularly alterations in *Firmicutes*, *Ruminococcaceae*, *Bacteroidia*, and *Actinobacteria*, may be associated with diabetic peripheral neuropathy and concomitant cognitive dysfunction (Huang et al., 2023). These findings indicate that gut microbial dysbiosis is closely linked to the onset and progression of PDN. Moreover, the role of the gut microbiota in regulating the glymphatic system has attracted increasing attention. For instance, the periodontal pathogen *Porphyromonas gingivalis* may induce cognitive impairment by disrupting gut microbial homeostasis, promoting neuroinflammation, and impairing glymphatic function (Chi et al., 2021). In addition, short-chain fatty acids (SCFAs), key metabolites of the gut microbiota, have been shown to modulate glial cell function by activating the tryptophan (Trp)–AhR–AQP4 signaling pathway and preventing the loss of AQP4 polarity in astrocytes (Lin et al., 2024). Although studies suggest a close link between the gut microbiota, PDN, and the glymphatic system, the specific mechanisms remain poorly understood. Therefore, this study establishes a PDN rat model using a high-fat, high-sugar diet combined with low-dose STZ intraperitoneal injection, a method that has been successfully validated multiple times in our research group (Li et al., 2024; Jia et al., 2024). Given that estrogen may influence glucose metabolism following STZ administration, male rats were specifically selected to eliminate potential hormonal confounding effects (Paschou and Papanas, 2019).

In the MRI experiments, all rats were anesthetized and maintained with sevoflurane. Considering the increased water demand in PDN rats, animals were allowed to recover and drink freely between scanning sessions to ensure physiological tolerance. Previous studies have shown that normal water intake does not significantly affect total brain water content (Meyers et al., 2016), suggesting minimal interference with glymphatic function assessment. However, accumulating evidence indicates that sevoflurane exposure can significantly alter gut microbiota composition and metabolite production in mice, particularly affecting taxa associated with cognition and health, such as *Streptococcus*, members of the *Lachnospiraceae* family, and *Pseudoflavonifractor*, as well as bile acid metabolism (Zhao et al., 2024; Liu et al., 2021). Furthermore, sevoflurane exposure has been found to impair the function of the glymphatic system in the central nervous system, which in turn affects the expression of AQP4 (Wang et al., 2024). Sevoflurane has also been shown to enhance glymphatic system clearance by upregulating AQP4 expression (Gao et al., 2019). To minimize these potential confounding effects, all animals were anesthetized under standardized conditions (2.5% sevoflurane with an oxygen flow rate of 2 L/min). In addition, the timing of subarachnoid puncture and tracer injection was strictly controlled, and MRI acquisition parameters, including scan timing and frequency, were kept consistent across all groups. Therefore, although sevoflurane may influence gut microbiota composition and AQP4 expression, the standardized experimental design ensured consistency across groups and minimized its potential impact on the study outcomes.

Ginkgolide B, a natural plant compound, has gained significant attention in recent years for its potential in neuroinflammation and immune modulation. Studies have shown that Ginkgolide B can alleviate neuroinflammation in a rat chronic constriction injury model by activating the PINK1-Parkin-mediated mitophagy pathway, thereby effectively reducing neuropathic pain (Liang et al., 2024). Furthermore, our previous research has also demonstrated that Ginkgolide B regulates the polarity of AQP4 by inhibiting MMP-9-mediated β-DG cleavage, thus improving spinal cord glymphatic system function. This mechanism may play an important role in alleviating PDN (Li et al., 2024). These findings suggest that Ginkgolide B has significant potential for the prevention and treatment of PDN. In addition to its role in regulating neuroinflammation, Ginkgolide B treatment has been shown to reverse the reduced abundance of *Lactobacilli* in Alzheimer’s disease mice, while increasing the abundance of microbiota such as *Bacteroidales*, *Muribaculaceae*, and *Alloprevotella*, thereby reconstructing the gut microbiota and improving cognitive function (Liu et al., 2021). Moreover, Ginkgolide B may alleviate hyperlipidemia, inflammation, atherosclerotic plaque formation, and gut barrier dysfunction induced by a high-fat diet by increasing the abundance of *Bacteroides* and reducing the abundance of *Helicobacter pylori* (Lv et al., 2021). Notably, the results of this study further confirm the positive effects of Ginkgolide B in anti-inflammatory, analgesic, and gut barrier repair. Overall, Ginkgolide B, through its multifaceted mechanisms, including the regulation of glymphatic system function, AQP4 expression and distribution, gut microbiota balance, and gut barrier integrity, offers novel therapeutic strategies for neuroprotective treatments and related diseases.

Our study found no consistent statistically significant differences in the α-diversity of the gut microbiota across the three rat groups. This suggests that certain samples did not exhibit significant changes in species richness or evenness. We hypothesize that the lack of significant differences in some α-diversity indices may be related to the specific type of disease being studied. Similar results have been observed in other studies investigating the relationship between PDN and gut microbiota (Huang et al., 2023; Xie et al., 2020a,b). This phenomenon may be attributed to the relatively subtle changes in species diversity of the gut microbiota in the context of diabetes or PDN, with the influence on health status being more indirect, mediated by alterations in community composition or changes in the abundance of specific microbes. It is also noteworthy that sample size and heterogeneity may impact the detection of changes in gut microbiota diversity, potentially leading to an underrepresentation of certain subtle changes. Nevertheless, β-diversity analysis, including PCoA and NMDS, revealed significant differences among the three groups, indicating notable variations in the gut microbiota community structure. This finding further suggests that changes in specific microbes or microbial communities may be closely associated with the onset and progression of the disease. Therefore, future research should focus on the role of specific microbes or microbial communities in disease, exploring their relationship with PDN and their potential clinical applications.

In this study, we found that the abundance of *Pseudomonadota* at the genus level was significantly higher in the group PDN compared to the group C and group PDN + GB. *Pseudomonadota* are a group of bacteria capable of surviving in hypoxic environments, and they are known to play significant roles in various pathological conditions. Previous studies have shown that the gut microbiota diversity in patients with necrotizing enterocolitis is significantly reduced, accompanied by overgrowth of opportunistic pathogens such as *Pseudomonas aeruginosa* and *Pseudomonas nosocomialis* (Tarracchini et al., 2021). Furthermore, an animal study indicated that the gut microbiota in intrauterine hypoxic rats is predominantly composed of *Pseudomonadota* (Sun et al., 2021). These findings suggest that an excessive increase in the abundance of *Pseudomonadota* may directly or indirectly promote the occurrence and development of intestinal inflammation. Further research has shown that the abundance of *Pseudomonadota* is also significantly elevated in patients with major depressive disorder and autism spectrum disorder, along with increased chronic low-grade inflammation and inflammatory factors (Amin et al., 2023; Lewandowska-Pietruszka et al., 2023). These results suggest a potential link between the increased abundance of *Pseudomonadota* and immune dysregulation. In line with our findings, the PDN rats exhibited marked inflammatory responses in both the gut and spinal cord, along with impaired intestinal barrier function and elevated levels of inflammatory factors in the spinal cord. Therefore, the over proliferation of *Pseudomonadota* in the group PDN may exacerbate intestinal inflammation and immune responses, leading to heightened central inflammation and further promoting the progression of neuropathy. The therapeutic effect of GB may involve the inhibition of *Pseudomonadota* proliferation, thereby alleviating the inflammatory response and exerting its therapeutic effect. At the genus level, we also observed that the abundance of *Phocaeicola*, *Bacteroides*, and *Escherichia* was higher in the group PDN compared to the group C. After GB treatment, the abundance of these genera showed a decreasing trend, suggesting that these genera may play an important role in the occurrence and development of PDN. Studies have pointed out that differences in gut microbiota composition between metabolic-associated fatty liver disease (MASLD) patients and healthy controls are mainly observed at the genus level, with MASLD patients’ gut microbiota predominantly consisting of *Phocaeicola* and *Bacteroides*, which are closely related to the risk of MASLD development (Lin et al., 2025). Additionally, in the gut microbiota of zinc-deficient children, the relative abundance of *Phocaeicola vulgatus* and *Bacteroides uniformis* is higher, and these strains exhibit higher glycoside hydrolase activity. The levels of sweeteners and tauroursodeoxycholic acid in the metabolites are elevated, with the latter possibly inducing leaky gut syndrome (Chai et al., 2024). This suggests that *Phocaeicola* and *Bacteroides* play key roles in carbohydrate digestion and metabolism. *Escherichia* is a common bacterium in the gut, and most strains (e.g., UPEC, EHEC) can secrete virulence factors (e.g., lipopolysaccharides and bacterial toxins), leading to urinary tract infections, food poisoning, and gastrointestinal diseases (Montalbetti et al., 2022; Nagao et al., 2024). In this study, the decrease in *Escherichia* abundance in the group PDN + GB indicates that GB may have an inhibitory effect on *Escherichia*, thereby alleviating intestinal inflammation. Taken together, these results suggest that GB may exert its potential therapeutic effect in diabetic neuropathy by modulating the gut microbiota, inhibiting the over proliferation of harmful microbes (such as *Pseudomonadota*), and promoting the proliferation of beneficial microbes (such as *Bacillus*).

The differential microbiota analysis results indicated that the gut microbiota of the group PDN was predominantly composed of *g_Enterocloster* and *g_Oscillibacter*, which could potentially exacerbate neuropathic symptoms. Relevant studies have shown that *Enterocloster bolteae* is more abundant in type 2 diabetes mellitus patients, and this bacterium may influence the onset of T2DM through its metabolic products or interactions with other gut microbiota (Park et al., 2023). In the gut microbiota of patients with diabetic nephropathy, depression, and constipation associated with type 2 diabetes, the abundance of *Oscillibacter* is significantly increased. Inhibiting its abundance can significantly reduce the levels of interleukin IL-1β, IL-6, and TNF-α, thereby alleviating intestinal inflammation and exerting potential therapeutic effects (Deng et al., 2022; Zhang et al., 2021). Therefore, these bacteria may promote the development of neuropathy by increasing intestinal permeability and chronic low-grade inflammation. In the group PDN + GB, the gut microbiota was primarily characterized by *g_Lactobacillus*, *g_Limosilactobacillus*, *g_Roseburia*, and *g_Faecalibacterium*. Studies have shown that *Lactobacillus* and *Limosilactobacillus* can not only improve obesity induced by energy metabolism imbalance through the production of SCFAs but also exhibit significant anti-hyperglycemic effects (Amabebe et al., 2020; Zhou et al., 2021). Additionally, *g_Roseburia* and *g_Faecalibacterium* are also capable of producing butyrate, which improves intestinal barrier function and reduces inflammation (Hays et al., 2024; Lee et al., 2020; Singh et al., 2022). Further studies have demonstrated that butyrate not only contributes to the maintenance and restoration of intestinal barrier integrity, but also plays a crucial role in modulating the gut–pain axis. Mechanistically, butyrate may alleviate pain associated with peripheral nerve injury through multiple pathways, including the regulation of gene expression via histone deacetylase (HDAC) inhibition, modulation of immune cell activity, and activation of G protein-coupled receptors (e.g., GPR41/43) on vagal afferent neurons, thereby influencing gut–brain communication (Zhang et al., 2021; Jiang et al., 2025; Bonomo et al., 2020; Kukkar et al., 2014). Notably, Fusco et al. reported that oral administration of sodium butyrate significantly ameliorated gut dysbiosis, reduced intestinal and spinal neuroinflammation, attenuated nociceptive sensitization, and promoted the restoration of butyrate-producing microbiota (Fusco et al., 2026). These findings suggest that the presence of *Lactobacillus* and *Limosilactobacillus* could favor proliferation of SCFA-producing bacteria via lactate production and pH modulation, while Roseburia and Faecalibacterium, as main butyrate producers, may help maintain intestinal barrier integrity and reduce inflammation.

Similarly, The observed correlations suggest that specific gut microbial taxa may contribute to the regulation of host metabolic status and neuropathic responses. Notably, several genera, including Dorea, Enterocloster, and Clostridium, were positively associated with body weight and PWT, while showing negative correlations with blood glucose, indicating a potential beneficial role in both metabolic homeostasis and pain modulation. Among these, Dorea, a known butyrate-producing genus, may exert its effects through SCFA-mediated pathways. Butyrate has been widely reported to regulate host energy metabolism, improve insulin sensitivity, and modulate nociceptive signaling via anti-inflammatory and neuroprotective mechanisms. Therefore, the positive association between Dorea and PWT observed in this study may reflect a role in alleviating neuropathic pain through SCFA-dependent gut–brain axis interactions. In contrast, genera such as Candida and Methanobrevibacter, which were negatively correlated with body weight and PWT but positively associated with blood glucose, may be linked to metabolic dysregulation and increased pain sensitivity. These taxa have been previously associated with gut dysbiosis and inflammatory responses, suggesting that their enrichment may contribute to both hyperglycemia and enhanced nociceptive sensitization. Furthermore, the negative correlations of Lactobacillus and Limosilactobacillus with blood glucose support their potential hypoglycemic effects. These genera are known to produce bioactive metabolites, including SCFAs and lactic acid, which can improve glucose metabolism, enhance intestinal barrier function, and reduce systemic inflammation. Their association with lower blood glucose levels in the present study is consistent with previous reports highlighting their beneficial metabolic roles. Collectively, these findings suggest that gut microbiota may influence host metabolic and neuropathic parameters through integrated mechanisms involving microbial metabolites, immune modulation, and gut–brain axis signaling. However, given the correlational nature of this analysis, further mechanistic studies are required to establish causal relationships.

Numerous studies have demonstrated that intestinal barrier dysfunction is a common feature in type 2 diabetes models, but this impairment is reversible. Restoring gut microbiota balance and rebuilding intestinal barrier integrity have been shown to mitigate the progression of type 2 diabetes (Chen et al., 2023; Yang et al., 2024). In addition, probiotic intervention in the PDN rat model has been shown to effectively repair the intestinal barrier, reduce serum levels of LPS and pro-inflammatory cytokines, protect the blood-nerve barrier, and ultimately slow the progression of neuropathy (Tesfaye et al., 2022). In this study, western blot analysis revealed significantly higher expression levels of intestinal barrier proteins (ZO-1, Occludin, and Claudin-1) in the group PDN + GB compared to the group C. These results suggest that GB may promote the expression of intestinal barrier proteins, thereby enhancing the integrity of the intestinal mechanical barrier. Further analysis also showed that GB treatment significantly altered the expression patterns of glycosyltransferases (GT8, GT101) and glycoside hydrolase families (GH36, GH31) in the gut microbiota of PDN rats. It has been well-established that glycosyltransferases and glycoside hydrolases in the mammalian gut microbiota play crucial roles in the synthesis and degradation of mannosides (Li et al., 2020). Mannosides are involved in several important biological functions, such as plant cell wall construction, cell signaling, and glycogen storage in certain parasites (Sharma et al., 2014; Zhang and Beverley, 2019). Under hyperglycemic conditions, we hypothesize that metabolic imbalances in the host and dysfunction in the gut microbiota may lead to a shift in microbial metabolism toward enhanced degradation of complex carbohydrates. This shift may result in significant inhibition of critical metabolic pathways such as amino acid metabolism, lipid metabolism, and energy metabolism, as observed in the group PDN. However, GB treatment significantly reversed these metabolic abnormalities, restoring the function of the affected metabolic pathways.

Together, these findings suggest that GB plays a pivotal role in alleviating PDN-related metabolic dysregulation and nerve damage. This is likely achieved by improving both the structural and functional aspects of the gut microbiota, ultimately contributing to the restoration of metabolic homeostasis and the prevention of further nerve damage.

This study elucidates the potential mechanisms by which GB ameliorates PDN, although several limitations remain. First, due to the resolution constraints of 3.0 T MRI and the frailty of PDN rats, the experimental duration was limited to only 5 h. This restriction hindered the comprehensive observation of the contrast agent’s metabolic dynamics within the spinal cord, thereby limiting a full understanding of GB’s therapeutic mechanism. Second, although metagenomic sequencing revealed differences in gut microbiota composition among the groups, metabolic profiling of the gut microbiome could not be performed due to experimental limitations. Future studies should integrate additional animal models and clinical data to further validate the associations and elucidate the roles of specific microbial taxa and their metabolites-particularly short-chain fatty acids-in the pathogenesis and progression of PDN. Moreover, the sample size of this study was relatively small, and analyses were confined to three groups: group PDN, group C, and group PDN + GB. The current experimental design did not include a control group treated with GB or a dysbiosis model treated with GB. Although our results demonstrated that GB significantly improved metabolic parameters and neuropathic pain in PDN mice, it remains unclear whether these effects are mediated through direct modulation of gut microbiota or secondary to systemic metabolic improvements. In particular, previous studies have shown that gut dysbiosis alone can induce neurosensory alterations, including increased pain sensitivity. Therefore, the absence of a dysbiotic group treated with GB limits our ability to determine whether GB exerts a direct regulatory effect on microbiota-driven neuropathic changes, independent of the diabetic context. In addition, without a control-treated group, it is difficult to fully exclude the possibility that GB may also influence host physiology under normal conditions, which would be important for assessing its specificity and safety. Therefore, future investigations should aim to increase the sample size and conduct multicenter studies to confirm GB’s regulatory effects on gut microbiota and assess its applicability across diverse clinical populations. And future research directions should also focus on exploring the therapeutic potential of specific microbial genera, especially the balance between short-chain fatty acid-producing bacteria and pathogenic taxa, in the treatment of diabetic neuropathic pain. Furthermore, detailed analyses of how the gut microbiota influences host immune responses, inflammatory processes, and neural damage are essential to clarify its mechanistic roles in PDN. Such insights would provide a theoretical basis for developing novel therapeutic strategies. The long-term efficacy of GB treatment also warrants further investigation, particularly across different stages of diabetic neuropathy.

## 5 Conclusion

In conclusion, Ginkgolide B treatment may exert peripheral neuroprotective effects by remodeling the gut microbiota composition, alleviating intestinal inflammation, enhancing the intestinal mechanical barrier, and promoting the recovery of the spinal cord glymphatic system. These findings may provide additional scientific evidence for the clinical application of Ginkgolide B in the prevention and treatment of PDN.

## Data Availability

The datasets presented in this study can be found in online repositories. The names of the repository/repositories and accession number(s) can be found in the article/Supplementary material. The metagenomic data presented in the study are deposited in the NCBI Sequence Read Archive (SRA) repository, accession number PRJNA1192041.

## Supplementary material

Supplementary Figure S1 Flowchart of the experiments. Group C: Healthy rats; Group PDN: PDN rats; Group PDN+GB: PDN rats were gavaged with ginkgolide B; Group PDN+ABX: Pseudo-sterile rats; Group PDN+ABX+GB: Pseudo-sterile rats were gavaged with ginkgolide B; MRI: Magnetic resonance imaging; HE&IF: Hematoxylin-eosin staining and immunofluorescence; WB: Western blot; ELISA: Enzyme-linked immunosorbent assay.

## Glossary

ABX: Antibiotic cocktail
ANOVA: One-way analysis of variance
AQP4: Aquaporin-4
BCA: Bicinchoninic acid assay
CNS: Central nervous system
ELISA: Enzyme-linked immunosorbent assay
FMT: Fecal microbiota transplantation
FSE: Fast spin echo
GB: Ginkgolide B
Gd-DTPA: Gadopentetic acid
HE: Hematoxylin–eosin staining
IF: Immunofluorescence
IPGTT: Intraperitoneal glucose tolerance test
ITT: Insulin tolerance test
LDA: Linear discriminant analysis
LEfSe: Linear discriminant analysis effect size
LPS: Lipopolysaccharides
MASLD: Metabolic-associated fatty liver disease
MRI: Magnetic resonance imaging
MRI SI: MRI signal intensity
NMDS: Non-metric multidimensional scaling
OD: Optical density
PBS: Phosphate-buffered saline
PCoA: Principal coordinates analysis
PDN: Painful diabetic neuropathy
PFA: Paraformaldehyde
PWT: Paw withdrawal thresholds
SCFAs: Short-chain fatty acids
STZ: Streptozotocin
WB: Western blot

## References

[ref1] Amabebe E. Robert F. O. Agbalalah T. Orubu E. S. F. (2020). Microbial dysbiosis-induced obesity: role of gut microbiota in homoeostasis of energy metabolism. Br. J. Nutr. 123, 1127–1137. doi: 10.1017/S0007114520000380 32008579

[ref2] Amin N. Liu J. Bonnechere B. MahmoudianDehkordi S. Arnold M. Batra R. . (2023). Interplay of metabolome and gut microbiome in individuals with major depressive disorder vs control individuals. JAMA Psychiatry 80, 597–609. doi: 10.1001/jamapsychiatry.2023.0685, 37074710 PMC10116384

[ref3] Aronson R. Chu L. Joseph N. Brown R. (2021). Prevalence and risk evaluation of diabetic complications of the foot among adults with type 1 and type 2 diabetes in a large Canadian population (PEDAL study). Can. J. Diabetes 45, 588–593. doi: 10.1016/j.jcjd.2020.11.011, 33582042

[ref4] Bonomo R. R. Cook T. M. Gavini C. K. White C. R. Jones J. R. Bovo E. . (2020). Fecal transplantation and butyrate improve neuropathic pain, modify immune cell profile, and gene expression in the PNS of obese mice. Proc. Natl. Acad. Sci. USA 117, 26482–26493. doi: 10.1073/pnas.2006065117, 33020290 PMC7584890

[ref5] Chai X. Chen X. Yan T. Zhao Q. Hu B. Jiang Z. . (2024). Intestinal barrier impairment induced by gut microbiome and its metabolites in school-age children with zinc deficiency. Nutrients 16:1289. doi: 10.3390/nu16091289, 38732540 PMC11085614

[ref6] Chen X. Chen C. Fu X. (2023). Dendrobium officinale polysaccharide alleviates type 2 diabetes mellitus by restoring gut microbiota and repairing intestinal barrier via the LPS/TLR4/TRIF/NF-kB Axis. J. Agric. Food Chem. 71, 11929–11940. doi: 10.1021/acs.jafc.3c02429 37526282

[ref7] Chi L. Cheng X. Lin L. Yang T. Sun J. Feng Y. . (2021). *Porphyromonas gingivalis*-induced cognitive impairment is associated with gut Dysbiosis, Neuroinflammation, and Glymphatic dysfunction. Front. Cell. Infect. Microbiol. 11:755925. doi: 10.3389/fcimb.2021.755925 34926316 PMC8672439

[ref8] DeFeudis F. V. Drieu K. (2000). *Ginkgo biloba* extract (EGb 761) and CNS functions: basic studies and clinical applications. Curr. Drug Targets 1, 25–58. doi: 10.2174/1389450003349380, 11475535

[ref9] Deng L. Yang Y. Xu G. (2022). Empagliflozin ameliorates type 2 diabetes mellitus-related diabetic nephropathy via altering the gut microbiota. Biochim. Biophys. Acta Mol. Cell Biol. Lipids 1867:159234. doi: 10.1016/j.bbalip.2022.159234, 36185030

[ref10] Dolo P. R. Yao L. Li C. Zhu X. Shi L. Widjaja J. (2018). Preserving duodenal-Jejunal (foregut) transit does not impair glucose tolerance and diabetes remission following gastric bypass in type 2 diabetes Sprague-Dawley rat model. Obes. Surg. 28, 1313–1320. doi: 10.1007/s11695-017-2985-y, 29098544

[ref11] Feldman E. L. Callaghan B. C. Pop-Busui R. Zochodne D. W. Wright D. E. Bennett D. L. . (2019). Diabetic neuropathy. Nat. Rev. Dis. Primers 5:42. doi: 10.1038/s41572-019-0092-1 31197183 PMC7096070

[ref12] Franzosa E. A. McIver L. J. Rahnavard G. Thompson L. R. Schirmer M. Weingart G. . (2018). Species-level functional profiling of metagenomes and metatranscriptomes. Nat. Methods 15, 962–968. doi: 10.1038/s41592-018-0176-y, 30377376 PMC6235447

[ref13] Furman B. L. (2021). Streptozotocin-induced diabetic models in mice and rats. Curr Protoc 1:e78. doi: 10.1002/cpz1.78 33905609

[ref14] Fusco A. Ricciardi F. Perrone M. Di Martino E. Limongelli R. Morace A. M. . (2026). Oral sodium butyrate supplementation acts on intestinal dysbiosis and resolves spinal cord neuroinflammation and nociceptive neuron sensitization. J Inflamm (Lond) 23:14. doi: 10.1186/s12950-026-00496-8, 41862960 PMC13126750

[ref15] Gao X. Ming J. Liu S. Lai B. Fang F. Cang J. (2019). Sevoflurane enhanced the clearance of Aβ1-40 in hippocampus under surgery via up-regulating AQP-4 expression in astrocyte. Life Sci. 221, 143–151. doi: 10.1016/j.lfs.2019.02.024, 30763576

[ref16] Guo K. Figueroa-Romero C. Noureldein M. Hinder L. M. Sakowski S. A. Rumora A. E. . (2023). Gut microbiota in a mouse model of obesity and peripheral neuropathy associated with plasma and nerve lipidomics and nerve transcriptomics. Microbiome 11:52. doi: 10.1186/s40168-022-01436-3, 36922895 PMC10015923

[ref17] Hablitz L. M. Nedergaard M. (2021). The Glymphatic system: a novel component of fundamental neurobiology. J. Neurosci. 41, 7698–7711. doi: 10.1523/JNEUROSCI.0619-21.2021, 34526407 PMC8603752

[ref18] Hays K. E. Pfaffinger J. M. Ryznar R. (2024). The interplay between gut microbiota, short-chain fatty acids, and implications for host health and disease. Gut Microbes 16:2393270. doi: 10.1080/19490976.2024.2393270, 39284033 PMC11407412

[ref19] Huang W. Lin Z. Sun A. Deng J. Manyande A. Xiang H. . (2023). The role of gut microbiota in diabetic peripheral neuropathy rats with cognitive dysfunction. Front. Microbiol. 14:1156591. doi: 10.3389/fmicb.2023.1156591 37266023 PMC10231493

[ref20] Huang L. Shi Y. Zhao L. (2021). Ginkgolide B alleviates learning and memory impairment in rats with vascular dementia by reducing Neuroinflammation via regulating NF-κB pathway. Front. Pharmacol. 12:676392. doi: 10.3389/fphar.2021.676392, 34220511 PMC8245850

[ref21] Iatcu C. O. Steen A. Covasa M. (2021). Gut microbiota and complications of Type-2 diabetes. Nutrients 14:166. doi: 10.3390/nu14010166, 35011044 PMC8747253

[ref22] Iliff J. J. Wang M. Liao Y. Plogg B. A. Peng W. Gundersen G. A. . (2012). A paravascular pathway facilitates CSF flow through the brain parenchyma and the clearance of interstitial solutes, including amyloid β. Sci. Transl. Med. 4:147ra111. doi: 10.1126/scitranslmed.3003748 PMC3551275 22896675

[ref23] Jia S. Y. Yin W. Q. Xu W. M. Li J. Yan W. Lin J. Y. (2024). Liquiritin ameliorates painful diabetic neuropathy in SD rats by inhibiting NLRP3-MMP-9-mediated reversal of aquaporin-4 polarity in the glymphatic system. Front. Pharmacol. 15:1436146. doi: 10.3389/fphar.2024.1436146, 39295943 PMC11408323

[ref24] Jiang Y. Huang Z. Sun W. Huang J. Xu Y. Liao Y. . (2025). *Roseburia intestinalis*-derived butyrate alleviates neuropathic pain. Cell Host Microbe 33, 104–118.e7. doi: 10.1016/j.chom.2024.11.013, 39706182

[ref25] Kress B. T. Iliff J. J. Xia M. Wang M. Wei H. S. Zeppenfeld D. . (2014). Impairment of paravascular clearance pathways in the aging brain. Ann. Neurol. 76, 845–861. doi: 10.1002/ana.24271, 25204284 PMC4245362

[ref26] Kukkar A. Singh N. Jaggi A. S. (2014). Attenuation of neuropathic pain by sodium butyrate in an experimental model of chronic constriction injury in rats. J. Formos. Med. Assoc. 113, 921–928. doi: 10.1016/j.jfma.2013.05.013, 23870713

[ref27] Langmead B. Salzberg S. L. (2012). Fast gapped-read alignment with bowtie 2. Nat. Methods 9, 357–359. doi: 10.1038/nmeth.1923, 22388286 PMC3322381

[ref28] Lee J. d'Aigle J. Atadja L. Quaicoe V. Honarpisheh P. Ganesh B. P. . (2020). Gut microbiota-derived short-chain fatty acids promote Poststroke recovery in aged mice. Circ. Res. 127, 453–465. doi: 10.1161/CIRCRESAHA.119.316448, 32354259 PMC7415518

[ref29] Lewandowska-Pietruszka Z. Figlerowicz M. Mazur-Melewska K. (2023). Microbiota in autism Spectrum disorder: a systematic review. Int. J. Mol. Sci. 24:16660. doi: 10.3390/ijms242316660, 38068995 PMC10706819

[ref30] Li J. Jia S. Song Y. Xu W. Lin J. (2024). Ginkgolide B can alleviate spinal cord glymphatic system dysfunction and provide neuroprotection in painful diabetic neuropathy rats by inhibiting matrix metalloproteinase-9. Neuropharmacology 250:109907. doi: 10.1016/j.neuropharm.2024.109907, 38492884

[ref31] Li A. Laville E. Tarquis L. Lombard V. Ropartz D. Terrapon N. . (2020). Analysis of the diversity of the glycoside hydrolase family 130 in mammal gut microbiomes reveals a novel mannoside-phosphorylase function. Microb. Genom. 6:6. doi: 10.1099/mgen.0.000404 PMC7660257 32667876

[ref32] Liang J. H. Yu H. Xia C. P. Zheng Y. H. Zhang Z. Chen Y. . (2024). Ginkgolide B effectively mitigates neuropathic pain by suppressing the activation of the NLRP3 inflammasome through the induction of mitophagy in rats. Biomed. Pharmacother. 177:117006. doi: 10.1016/j.biopha.2024.117006, 38908197

[ref33] Lin X. Peng Y. Guo Z. He W. Guo W. Feng J. . (2024). Short-chain fatty acids suppresses astrocyte activation by amplifying Trp-AhR-AQP4 signaling in experimental autoimmune encephalomyelitis mice. Cell. Mol. Life Sci. 81:293. doi: 10.1007/s00018-024-05332-x, 38976012 PMC11335219

[ref34] Lin Y. C. Wu C. C. Li Y. E. Chen C. L. Lin C. R. Ni Y. H. (2025). Full-length 16S rRNA sequencing reveals gut microbiome signatures predictive of MASLD in children with obesity. BMC Microbiol. 25:146. doi: 10.1186/s12866-025-03849-0, 40091070 PMC11912586

[ref35] Liu S. Lam M. A. Sial A. Hemley S. J. Bilston L. E. Stoodley M. A. (2018). Fluid outflow in the rat spinal cord: the role of perivascular and paravascular pathways. Fluids Barriers CNS 15:13. doi: 10.1186/s12987-018-0098-1, 29704892 PMC5924677

[ref36] Liu M. Song S. Chen Q. Sun J. Chu W. Zhang Y. . (2021). Gut microbiota mediates cognitive impairment in young mice after multiple neonatal exposures to sevoflurane. Aging (Albany NY) 13, 16733–16748. doi: 10.18632/aging.203193, 34182544 PMC8266337

[ref37] Liu J. Ye T. Zhang Y. Zhang R. Kong Y. Zhang Y. . (2021). Protective effect of Ginkgolide B against cognitive impairment in mice via regulation of gut microbiota. J. Agric. Food Chem. 69, 12230–12240. doi: 10.1021/acs.jafc.1c05038, 34633804

[ref38] Lv Z. Shan X. Tu Q. Wang J. Chen J. Yang Y. (2021). Ginkgolide B treatment regulated intestinal flora to improve high-fat diet induced atherosclerosis in ApoE(−/−) mice. Biomed. Pharmacother. 134:111100. doi: 10.1016/j.biopha.2020.111100, 33341056

[ref39] Ma P. Mo R. Liao H. Qiu C. Wu G. Yang C. . (2022). Gut microbiota depletion by antibiotics ameliorates somatic neuropathic pain induced by nerve injury, chemotherapy, and diabetes in mice. J. Neuroinflammation 19:169. doi: 10.1186/s12974-022-02523-w, 35764988 PMC9237999

[ref40] Mandal S. Van Treuren W. White R. A. Eggesbø M. Knight R. Peddada S. D. (2015). Analysis of composition of microbiomes: a novel method for studying microbial composition. Microb. Ecol. Health Dis. 26:27663. doi: 10.3402/mehd.v26.27663 26028277 PMC4450248

[ref41] Meyers S. M. Tam R. Lee J. S. Kolind S. H. Vavasour I. M. Mackie E. . (2016). Does hydration status affect MRI measures of brain volume or water content? J. Magn. Reson. Imaging 44, 296–304. doi: 10.1002/jmri.25168 26825048

[ref42] Montalbetti N. Dalghi M. G. Bastacky S. I. Clayton D. R. Ruiz W. G. Apodaca G. . (2022). Bladder infection with uropathogenic *Escherichia coli* increases the excitability of afferent neurons. Am. J. Physiol. Renal Physiol. 322, F1–f13. doi: 10.1152/ajprenal.00167.2021, 34779263 PMC8698541

[ref43] Nagao I. Kawasaki M. Goyama T. Kim H. J. Call D. R. Ambrosini Y. M. (2024). Enterohemorrhagic *Escherichia coli* (EHEC) disrupts intestinal barrier integrity in translational canine stem cell-derived monolayers. Microbiol. Spectrum 12:e0096124. doi: 10.1128/spectrum.00961-24, 39162490 PMC11448187

[ref44] Park S. Zhang T. Kang S. (2023). Fecal microbiota composition, their interactions, and metagenome function in US adults with type 2 diabetes according to Enterotypes. Int. J. Mol. Sci. 24:9533. doi: 10.3390/ijms24119533 37298483 PMC10253423

[ref45] Paschou S. A. Papanas N. (2019). Type 2 diabetes mellitus and menopausal hormone therapy: an update. Diabetes Ther 10, 2313–2320. doi: 10.1007/s13300-019-00695-y 31549295 PMC6848654

[ref46] Peng S. Liu J. Liang C. Yang L. Wang G. (2023). Aquaporin-4 in glymphatic system, and its implication for central nervous system disorders. Neurobiol. Dis. 179:106035. doi: 10.1016/j.nbd.2023.106035, 36796590

[ref47] Qi H. Mariager C. Lindhardt J. Nielsen P. M. Stødkilde-Jørgensen H. Laustsen C. (2018). Effects of anesthesia on renal function and metabolism in rats assessed by hyperpolarized MRI. Magn. Reson. Med. 80, 2073–2080. doi: 10.1002/mrm.27165, 29520870

[ref48] Rakoff-Nahoum S. Paglino J. Eslami-Varzaneh F. Edberg S. Medzhitov R. (2004). Recognition of commensal microflora by toll-like receptors is required for intestinal homeostasis. Cell 118, 229–241. doi: 10.1016/j.cell.2004.07.002, 15260992

[ref49] Ren M. Xia Y. Pan H. Zhou X. Yu M. Ji F. (2025). Duodenal-jejunal bypass ameliorates MASLD in rats by regulating gut microbiota and bile acid metabolism through FXR pathways. Hepatol. Commun. 9:9. doi: 10.1097/HC9.0000000000000615 PMC11737483 39813598

[ref50] Sabico S. Al-Mashharawi A. Al-Daghri N. M. Wani K. Amer O. E. Hussain D. S. . (2019). Effects of a 6-month multi-strain probiotics supplementation in endotoxemic, inflammatory and cardiometabolic status of T2DM patients: a randomized, double-blind, placebo-controlled trial. Clin. Nutr. 38, 1561–1569. doi: 10.1016/j.clnu.2018.08.009, 30170781

[ref51] Salminen A. (2023). Activation of aryl hydrocarbon receptor (AhR) in Alzheimer's disease: role of tryptophan metabolites generated by gut host-microbiota. J. Mol. Med. (Berl) 101, 201–222. doi: 10.1007/s00109-023-02289-5, 36757399 PMC10036442

[ref52] Sharma V. Ichikawa M. Freeze H. H. (2014). Mannose metabolism: more than meets the eye. Biochem. Biophys. Res. Commun. 453, 220–228. doi: 10.1016/j.bbrc.2014.06.021, 24931670 PMC4252654

[ref53] Singh V. Lee G. Son H. Koh H. Kim E. S. Unno T. . (2022). Butyrate producers, "the sentinel of gut": their intestinal significance with and beyond butyrate, and prospective use as microbial therapeutics. Front. Microbiol. 13:1103836. doi: 10.3389/fmicb.2022.1103836 36713166 PMC9877435

[ref54] Sloan G. Shillo P. Selvarajah D. Wu J. Wilkinson I. D. Tracey I. . (2018). A new look at painful diabetic neuropathy. Diabetes Res. Clin. Pract. 144, 177–191. doi: 10.1016/j.diabres.2018.08.020 30201394

[ref55] Su X. Wu B. Zhang W. Ji Y. H. Wang Q. Tan Z. Y. (2019). Inhibitory effects of Columbianadin on nociceptive behaviors in a neuropathic pain model, and on voltage-gated calcium currents in dorsal root ganglion neurons in mice. Front. Pharmacol. 10:1522. doi: 10.3389/fphar.2019.01522 31998126 PMC6970200

[ref56] Sun Y. Li L. Song J. Mao W. Xiao K. Jiang C. (2021). Intrauterine hypoxia changed the colonization of the gut microbiota in newborn rats. Front. Pediatr. 9:675022. doi: 10.3389/fped.2021.675022, 33981656 PMC8107277

[ref57] Sun H. Saeedi P. Karuranga S. Pinkepank M. Ogurtsova K. Duncan B. B. . (2022). IDF diabetes atlas: global, regional and country-level diabetes prevalence estimates for 2021 and projections for 2045. Diabetes Res. Clin. Pract. 183:109119. doi: 10.1016/j.diabres.2021.109119, 34879977 PMC11057359

[ref58] Tarracchini C. Milani C. Longhi G. Fontana F. Mancabelli L. Pintus R. . (2021). Unraveling the microbiome of necrotizing Enterocolitis: insights in novel microbial and Metabolomic biomarkers. Microbiol. Spectrum 9:e0117621. doi: 10.1128/Spectrum.01176-21, 34704805 PMC8549755

[ref59] Tesfaye S. Sloan G. Petrie J. White D. Bradburn M. Young T. . (2022). Optimal pharmacotherapy pathway in adults with diabetic peripheral neuropathic pain: the OPTION-DM RCT. Health Technol. Assess. 26, 1–100. doi: 10.3310/RXUO6757, 36259684 PMC9589396

[ref60] Tokhi A. Ahmed Z. Arif M. Rehman N. U. Sheibani V. Sewell R. D. E. . (2023). Effects of 1-methyl-1, 2, 3, 4-tetrahydroisoquinoline on a diabetic neuropathic pain model. Front. Pharmacol. 14:1128496. doi: 10.3389/fphar.2023.1128496, 37033637 PMC10073420

[ref61] Wang Y. Ye X. Ding D. Lu Y. (2020). Characteristics of the intestinal flora in patients with peripheral neuropathy associated with type 2 diabetes. J. Int. Med. Res. 48:300060520936806. doi: 10.1177/0300060520936806, 32938282 PMC7503028

[ref62] Wang S. Yu X. Cheng L. Ren W. Wen G. Wu X. . (2024). Dexmedetomidine improves the circulatory dysfunction of the glymphatic system induced by sevoflurane through the PI3K/AKT/ΔFosB/AQP4 pathway in young mice. Cell Death Dis. 15:448. doi: 10.1038/s41419-024-06845-w, 38918408 PMC11199640

[ref63] Watada S. Yu Y. M. Fischman A. J. Kurihara T. Shen C. A. Tompkins R. G. . (2014). Evaluation of intragastric vs intraperitoneal glucose tolerance tests in the evaluation of insulin resistance in a rodent model of burn injury and glucagon-like polypeptide-1 treatment. J. Burn Care Res. 35, e66–e72. doi: 10.1097/BCR.0b013e31828a8ede, 23511296 PMC4053788

[ref64] Xie J. Song W. Liang X. Zhang Q. Shi Y. Liu W. . (2020a). Protective effect of quercetin on streptozotocin-induced diabetic peripheral neuropathy rats through modulating gut microbiota and reactive oxygen species level. Biomed. Pharmacother. 127:110147. doi: 10.1016/j.biopha.2020.110147, 32559841

[ref65] Xie J. Song W. Liang X. Zhang Q. Shi Y. Liu W. . (2020b). Jinmaitong ameliorates diabetic peripheral neuropathy in streptozotocin-induced diabetic rats by modulating gut microbiota and neuregulin 1. Aging (Albany NY) 12, 17436–17458. doi: 10.18632/aging.103750, 32920546 PMC7521543

[ref66] Xu M. Hao J. Qi Y. Wu B. Li R. Yang X. . (2024). Causal effects of gut microbiota on diabetic neuropathy: a two-sample Mendelian randomization study. Front. Endocrinol. (Lausanne) 15:1388927. doi: 10.3389/fendo.2024.1388927, 39157679 PMC11329939

[ref67] Yang H. Li G. P. Liu Q. Zong S. B. Li L. Xu Z. L. . (2021). Neuroprotective effects of Ginkgolide B in focal cerebral ischemia through selective activation of prostaglandin E2 receptor EP4 and the downstream transactivation of epidermal growth factor receptor. Phytother. Res. 35, 2727–2744. doi: 10.1002/ptr.7018, 33452698

[ref68] Yang J. Wang J. Wu W. Su C. Wu Y. Li Q. (2024). Xylooligosaccharides ameliorate insulin resistance by increasing Akkermansia muciniphila and improving intestinal barrier dysfunction in gestational diabetes mellitus mice. Food Funct. 15, 3122–3129. doi: 10.1039/D3FO04681H, 38426554

[ref69] Yang J. Yang X. Wu G. Huang F. Shi X. Wei W. . (2023). Gut microbiota modulate distal symmetric polyneuropathy in patients with diabetes. Cell Metab. 35:e7, 1548–1562. doi: 10.1016/j.cmet.2023.06.010 37451270

[ref70] Zhang K. Beverley S. M. (2019). Mannogen-ing central carbon metabolism by Leishmania. Trends Parasitol. 35, 947–949. doi: 10.1016/j.pt.2019.10.001, 31662278 PMC8018590

[ref71] Zhang X. Chen S. Zhang M. Ren F. Ren Y. Li Y. . (2021). Effects of fermented Milk containing Lacticaseibacillus paracasei strain Shirota on constipation in patients with depression: a randomized, double-blind, placebo-controlled trial. Nutrients 14:13. doi: 10.3390/nu14010013, 34209804 PMC8308326

[ref72] Zhang L. Liu C. Jiang Q. Yin Y. (2021). Butyrate in energy metabolism: there is still more to learn. Trends Endocrinol. Metab. 32, 159–169. doi: 10.1016/j.tem.2020.12.003, 33461886

[ref73] Zhao Y. Ma S. Liang L. Cao S. Fan Z. He D. . (2024). Gut microbiota-metabolite-brain Axis reconstitution reverses Sevoflurane-induced social and synaptic deficits in neonatal mice. Research (Wash D C) 7:0482. doi: 10.34133/research.0482 39301264 PMC11411162

[ref74] Zhou X. Shang G. S. Tan Q. He Q. Tan X. Park K. Y. . (2021). Effect of *Lactobacillus fermentum* TKSN041 on improving streptozotocin-induced type 2 diabetes in rats. Food Funct. 12, 7938–7953. doi: 10.1039/D1FO01571K, 34251007

