# Supplementary figures and images for: From gut to spinal cord glymphatic: Ginkgolide B’s multifaceted approach to alleviating painful diabetic neuropathy
Source: Front Microbiol. 2026 May 13;17:1833646. doi: 10.3389/fmicb.2026.1833646 (PMC13224466; doi:10.3389/fmicb.2026.1833646)

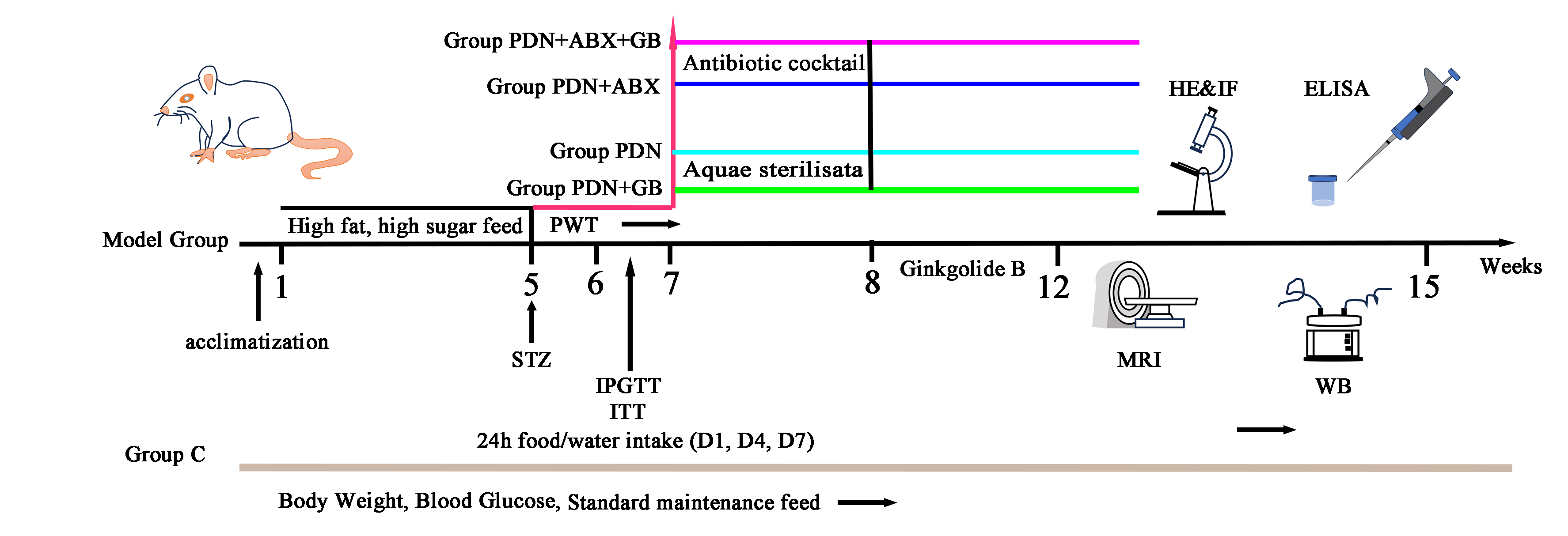

Supplement: Supplementary Figure S1 — Flowchart of the experiments. Group C: Healthy rats; Group PDN: PDN rats; Group PDN+GB: PDN rats were gavaged with ginkgolide B; Group PDN+ABX: Pseudo-sterile rats; Group PDN+ABX+GB: Pseudo-sterile rats were gavaged with ginkgolide B; MRI: Magnetic resonance imaging; HE&IF: Hematoxylin-eosin staining and immunofluorescence; WB: Western blot; ELISA: Enzyme-linked immunosorbent assay. [file Image_1.TIF]
